# Progress in Simulation Studies of Insulin Structure and Function

**DOI:** 10.3389/fendo.2022.908724

**Published:** 2022-06-20

**Authors:** Biswajit Gorai, Harish Vashisth

**Affiliations:** Department of Chemical Engineering, University of New Hampshire, Durham, NH, United States

**Keywords:** insulin, hormone, peptide, diabetes, insulin receptor, molecular dynamics, computer simulation

## Abstract

Insulin is a peptide hormone known for chiefly regulating glucose level in blood among several other metabolic processes. Insulin remains the most effective drug for treating diabetes mellitus. Insulin is synthesized in the pancreatic β-cells where it exists in a compact hexameric architecture although its biologically active form is monomeric. Insulin exhibits a sequence of conformational variations during the transition from the hexamer state to its biologically-active monomer state. The structural transitions and the mechanism of action of insulin have been investigated using several experimental and computational methods. This review primarily highlights the contributions of molecular dynamics (MD) simulations in elucidating the atomic-level details of conformational dynamics in insulin, where the structure of the hormone has been probed as a monomer, dimer, and hexamer. The effect of solvent, pH, temperature, and pressure have been probed at the microscopic scale. Given the focus of this review on the structure of the hormone, simulation studies involving interactions between the hormone and its receptor are only briefly highlighted, and studies on other related peptides (e.g., insulin-like growth factors) are not discussed. However, the review highlights conformational dynamics underlying the activities of reported insulin analogs and mimetics. The future prospects for computational methods in developing promising synthetic insulin analogs are also briefly highlighted.

## 1 Introduction

The discovery of insulin is a remarkable achievement in the history of biomedicine where insulin is noted as a model protein in several scientific findings (1–3). The enormous research conducted on the hormone insulin has enriched our knowledge of the protein synthesis, protein chemistry, structural biology, cell biology, and physiology. Insulin was first identified and isolated by Drs. Fredrick Banting and Charles Best with the support of Professor John MacLeod and James Collip from the pancreatic extract of dogs (4–7). Insulin was first used about 100 years ago to save a 14 year old boy who was suffering from type 1 diabetes mellitus (2). The discovery of animal-sourced insulin is one of the revolutionary breakthroughs in molecular medicine and it is also known as a ‘miracle drug’ (8). Bovine insulin was the first protein to be sequenced by the British biochemist Frederick Sanger (9) for which he was awarded the 1958 Noble Prize in Chemistry. Eight years later, insulin was the first protein to be chemically synthesized in the lab (10–12). A significant breakthrough by Genentech laboratory was achieved when insulin became the first human protein to be expressed in *E. coli* by using the recombinant DNA technology (13). Though insulin is not the first protein to have its structure determined, it is capped as the first protein hormone whose three-dimensional structure in dimeric and hexameric forms was determined using X-ray crystallography at a high resolution (14). While the focus of this review article is on modeling and simulation studies, the research findings from experiments on the synthesis, structure, conformational changes, physiological applications, and interactions with the receptor are summarized in several published reviews (2, 3, 5–7, 15–29).

Insulin is a small peptide hormone with 51 residues which regulates the glucose concentration in the bloodstream (7, 16). Insulin is secreted by the pancreatic *β*-cells to normalize the elevated glucose levels in the blood (7). Insulin stimulates a cascade of intracellular signaling processes which results in the intake of glucose in the tissues through different classes of eukaryotic sugar transporters (30–32). Glucose provides energy for cell functioning or is stored as fat. Abnormalities in insulin secretion due to the destruction of *β*-cells or damaged pancreas or insulin resistance reduce the level of insulin in the blood and hamper the glucose intake in cells. Insufficient insulin in the bloodstream leads to type 1 or type 2 diabetes mellitus (33, 34).

**Figure 1:**
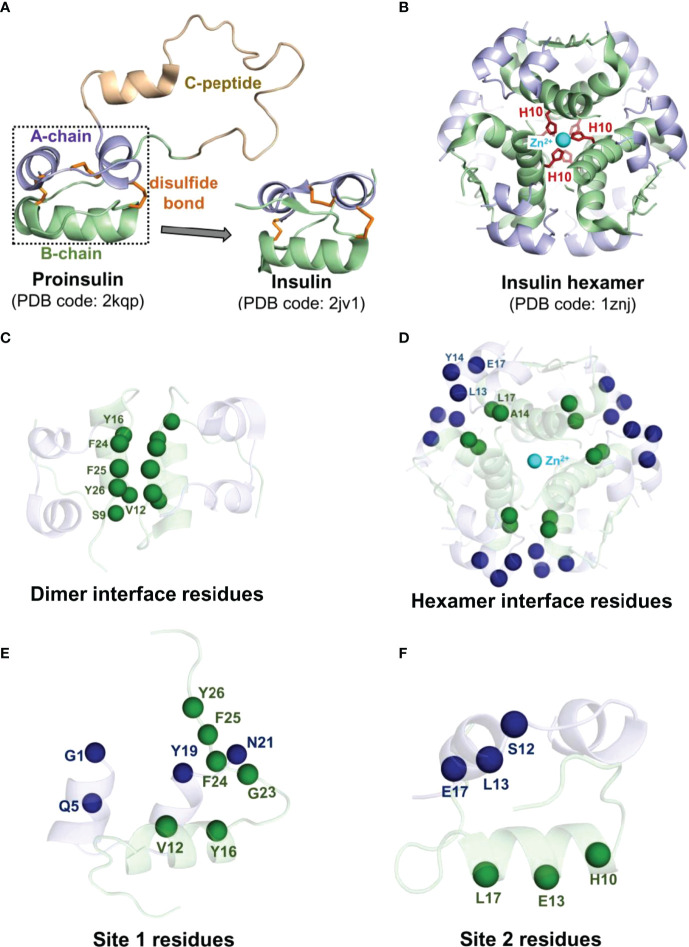
Structural features of the hormone insulin. **(A)** Shown is the structure of proinsulin consisting of the A-chain (light blue), the B-chain (pale green), and the C-peptide (light brown) connecting the A and B chains. **(B)** The R_6_ insulin hexamer with two zinc ions (cyan spheres) and coordinated histidine residues (red sticks) of the B-chain are depicted. The disulfide bonds are represented in orange sticks. The C*_α_* atoms of the interface residues engaged in the dimer interface **(C)** and the hexamer interface **(D)** of each insulin are depicted as spheres in the same color as each chain. The C*_α_* atoms of the site 1 **(E)** and site 2 **(F)** residues of insulin are depicted as spheres. The residues belonging to the A-chain and the B-chain of insulin are represented in blue and green spheres, respectively.

Insulin produced in the *β*-cells of the islets of Langerhans, termed proinsulin, includes a C-peptide (35 residues) connecting the A- and B-chains (**Figure 1A**) (35, 36). Once the proinsulin moves from the endoplasmic reticulum to the Golgi apparatus of the *β*-cell, the C-peptide is cleaved by specific endopeptidases resulting in the mature form of insulin (37). The mature form of insulin is a peptide hormone consisting of an A-chain (21 residues) and a B-chain (30 residues) held together by two inter-chain disulfide bonds (A7:B7 and A20:B19) and an intra-chain disulfide bond (A6:A11) in the A-chain. Moreover, insulin self-associates into a hexameric architecture due to a high concentration of zinc ions (Zn^2+^) in the Golgi apparatus (38). Insulin hexamer is the storage form of insulin where Zn^2+^ ions stabilize the hexamer structure. Each Zn^2+^ ion establishes interactions with every His10 residue of the B-chain of three insulin subunits (**Figure 1B**). Insulin hexamer initially dissociates to a dimer and then to a monomer after reaching the blood circulation. The residues in the dimer (**Figure 1C**) and hexamer (**Figure 1D**) forming interfaces are distinct. The C-terminal residues of the B-chain (PheB24-TyrB26) mainly contribute to the dimerization of insulin. Along with Zn^2+^ ions, the residues AlaA14, LeuA17, LeuB13, TyrB14, and GluB17 help in maintaining the hexameric architecture of insulin. However, it must be noted that the insulin monomer is the biologically active form of hormone that binds to its cognate receptor, the insulin receptor (IR), and initiates complex signaling pathways underlying glucose homeostasis (39). Insulin interacts with two distinct sites on IR *via* two surfaces known as “site 1” and “site 2”. The site 1 binding surface of insulin is comprised of residues GlyA1, GlnA5, TyrA19, AsnA21, ValB12, TyrB16, PheB24, PheB25, and TyrB26 (**Figure 1E**), while the site 2 surface is composed of residues SerA12, LeuA13, GluA17, HisB10, GluB13, and LeuB17 (**Figure 1F**) (39, 40).

**Figure 2:**
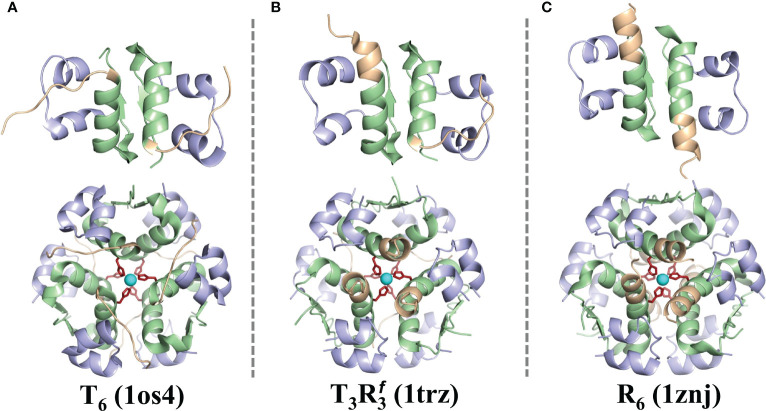
The T_6_, T_3_ ${\text{R}}_{3}^{\text{f}}$ , and R_6_ forms of insulin. The cartoon representations of the structures of dimeric and hexameric forms of insulin are shown in the T_6_ **(A)**, T_3_ ${\text{R}}_{3}^{\text{f}}$ **(B)**, and the R_6_ **(C)** states. The key residues of the B-chain (B1-B8) that exhibit a distinct structural transition from an extended to a helical conformer are depicted in light brown. cf. **Figure 1** for color scheme and other details.

The mature form of the hormone is comprised of three *α*-helices: the A-chain contains two short helices (residues Gly1 to Ser9 and residues Leu13 to Tyr19) and the B-chain contains a single large helix (residues Phe1 to Cys19). However, some residues in the N-terminus of the B-chain (residues Phe1 to Gly8) in the insulin hexamer exhibit structural transitioning between a helix and an unstructured fragment in different solvent environments (41–43). Specifically, the first eight residues of the B-chain of each insulin monomer in the hexamer can take an unstructured conformation, which is designated as the T_6_ state (**Figure 2A**), especially when the crystals are obtained at a low chloride ion concentration (44). At higher concentrations of singly charged inorganic anions (Cl^−^, I^−^, ${\text{N}}_{3}^{-}$ , SCN^−^, etc.), the T_3_ ${\text{R}}_{3}^{\text{f}}$ state (**Figure 2B**) is mostly populated with the unstructured B-chain in three insulin monomers, while the residues B4-B19 remain *α* helical in remaining three insulin monomers. In the presence of small cyclic alcohols (like phenol, cresol, resorcinol, hexanol, and pentanol), the insulin hexamer exhibits the R_6_ state with the B-chain residues (Phe1 to Cys19) of each monomer adapting an *α* helical conformation (**Figure 2C**). The T-state of insulin is prevalent in monomer, dimer, and IR binding states. The physiological relevance of the T↔R transition in the N-terminal residues of the B-chain of insulin still remains unclear (43).

Along with the experimental techniques, molecular modeling and simulation techniques have also provided a detailed picture of the atomic motions in insulin. Specifically, molecular dynamics (MD) simulations, which predict the motion of each atom as a function of time by integrating the equation of motion, have been a key computational method to probe biomolecular dynamics. In an MD simulation, the spatial positions of the atoms in a biomolecule are governed by bonded and non-bonded interactions which are defined by an interatomic potential energy function, often termed as the force-field (45). MD-based simulated annealing is also widely used in combination with the experimental techniques including nuclear magnetic resonance (NMR) spectroscopy, X-ray crystallography, and Cryoelectron microscopy (cryo-EM) to predict and refine biomolecular structures. All three techniques (NMR, X-ray, cryo-EM) have been used for structure determination of the insulin family of proteins (46–55). An energetically reasonable structure from these techniques provides a good starting conformer for performing extensive MD simulations. Additionally, MD simulations are significantly used to address specific problems in protein folding, function, dynamics, interactions, and drug design.

## 2 Review of Simulation Studies (1985 - To Date)

**Figure 3:**
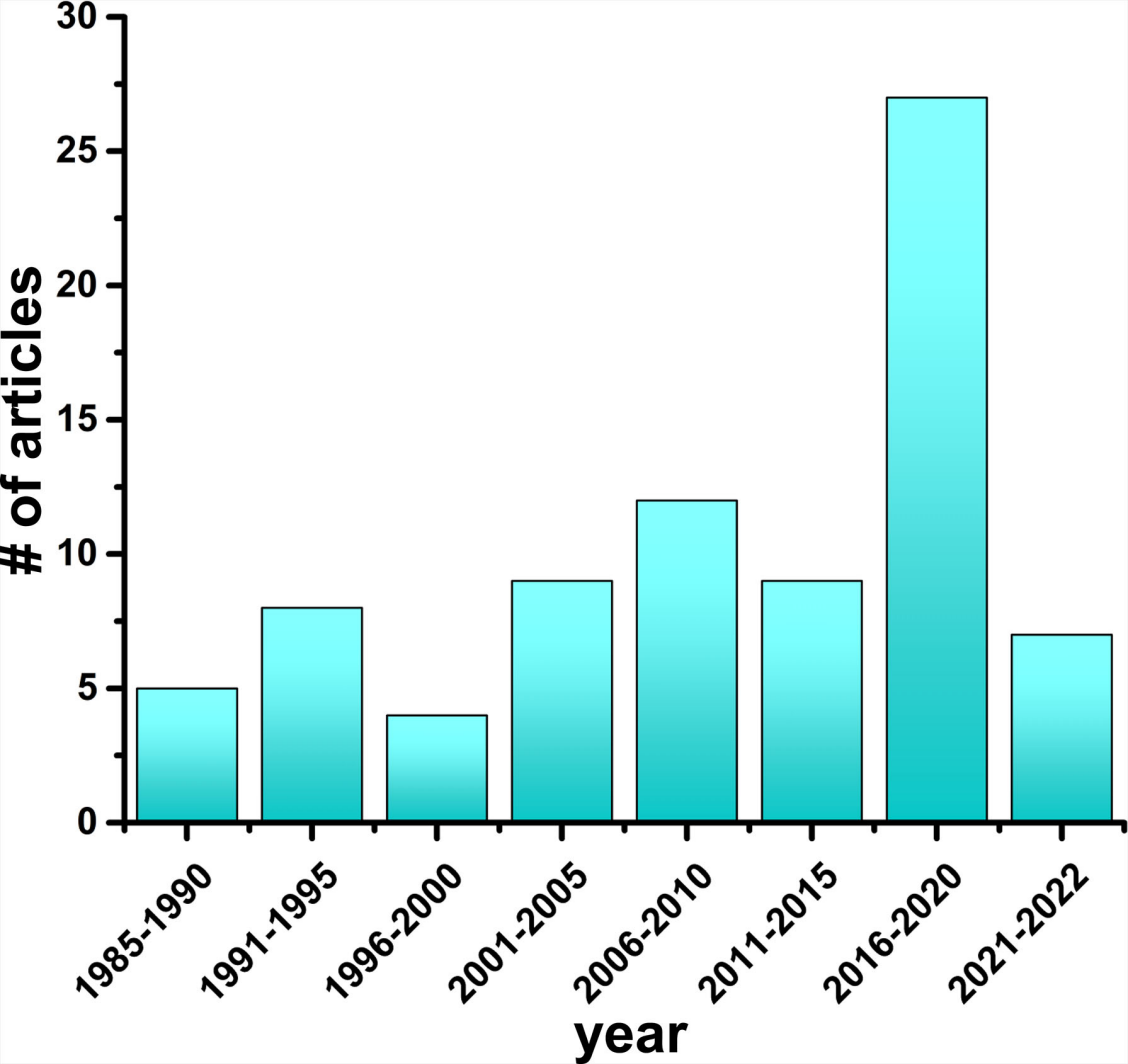
Simulation studies on the hormone insulin (1985–2022). The number of research articles on MD simulations of insulin published every 5-year time interval is depicted.

In this review, we explicitly focus on highlighting the findings from molecular simulation studies probing the conformational dynamics of insulin. Starting with studies published in 1985 to the most up to date work, we have organized studies into 5-year periods, with 7 complete periods and the 8^th^ an ongoing period. As of February 2022, we found a total of 81 published studies (**Figure 3**) that have implemented molecular modeling techniques to study the structure of the hormone insulin (**Table 1**). The advantages and limitations of simulation approaches as well as future opportunities and challenges in studying insulin are also discussed. To gain a comprehensive understanding of the fundamentals of MD simulations and their applications, we refer interested readers to previous comprehensive reviews (21, 134–143).

**Table 1:** A chronological summary of simulation studies on different forms of the hormone insulin (M, monomer; D, dimer; and H, hexamer).

#	System studied	PDB code (organism)	Simulation parameters	Reference
1	M, D, H	n/a (pig)	EM, vacuum, n/a	(56)
2	M	n/a (pig)	EM, vacuum, n/a	(57)
3	M	n/a (pig)	120 ps, vacuum, 300 K	(58)
4	DPI	n/a	31 ps, SPC, 291 K	(59)
5	M, D	n/a (pig)	100 ps, SPC, 300 K	(60)
6	H	n/a (pig)	n/a, n/a, 300 K	(61)
7	DPI	n/a (pig)	30 ps, vacuum, 300 K	(46)
8	M, D	1ins (pig)	100 ps, SPC, 300 K	(62)
9	M	n/a (pig)	TEM, vacuum, n/a	(63)
10	M	n/a (pig)	200 ps TMD, vacuum, 300 K	(64)
11	M, D, DASI	n/a (cow, pig)	500 ps, spc, 300 K	(65)
12	M, D	n/a (pig)	EM, vacuum, 300 K	(66)
13	M	n/a (pig)	200 ps TMD, vacuum, 300 K	(67)
14	H	n/a (pig)	200 ps TMD, vacuum, 300 K	(68)
15	H	n/a (pig)	12 ps SA, n/a, 300 K	(69)
16	B-chain	3ins (pig)	96 ps, SPC, 300 K	(70)
17	M	9ins (pig)	200 ps, TIPS3P, 298 K	(71)
18	D	4ins (pig)	600 ps, SPC/E, 300 K	(72)
19	D	4ins (pig)	600 ps, SPC/E, 300 K	(73)
20	H	1znj (human)	600 ps, SPC, 300 K	(74)
21	H	n/a (human)	1 ns, SPC, 300 K	(75)
22	M	4ins (pig)	1 ns, TIP3P, 300 K	(76)
23	M, D	4ins, 1b9e (pig, human)	10 ns, TIP3P, 300 K	(77)
24	M, B-chain	1zni (pig)	2 ns, TIP3P, 400 K	(78)
25	B-chain	1zni (pig)	10 ns, TIP3P, 400 K	(79)
26	D	4ins (pig)	5 ns, TIP3P, 400 K	(80)
27	D	4ins (pig)	3.5 ns SMD, SPC, 300 K	(81)
28	M	4ins (pig)	10 ns CMEPS, TIP3P, 300 K	(82)
29	B-chain	1zni (pig)	20 ns, TIP3P, 400 K	(83)
30	B-chain	1zni (pig)	10 ns, TIP3P, 300 K	(84)
31	M, B-chain	1zni (pig)	10 ns, TIP3P, 400 K	(85)
32	B-chain	1zni (pig)	10 ns, (SPC, SPC/E, TIP3P, TIP4P, and TIP4P-Ew), 300 K	(86)
33	H	1znj (human)	1 ns RAMD/SMD, TIP3P, 300 K	(87)
34	B-chain	1zni (pig)	96 ns BE-META, TIP3P, 300 K	(88)
35	B-chain	1zeh (human)	6 ns, SPC, 300 K	(89)
36	M	2bn3 (cow)	95 ns, TIP4P, 313 K	(90)
37	M	1zeh (human)	10 ns, TIP3P, 298 K	(91)
38	B-chain	1zni (pig)	US and BE-META, n/a, n/a	(92)
39	PEG-M	4ins (pig)	50 ns, SPC, 300 K	(93)
40	M	2a3g (cow)	10 ns, n/a, 300 K	(94)
41	M	1mso (human)	50 ns, TIP3P, 300 K	(95)
42	M, DPI	2zp6, 1his (cow, human)	15 ns, TIP3P, 500 K	(96)
43	M	9ins (pig)	20 ns, SPC/E, 300 K	(97)
44	ProI	2kqp (human)	200 ns, TIP3P, 310 K	(98)
45	M	3w7y (human)	100 ns, TIP3P, 338 K	(99)
46	M	2g4m (pig)	120 ns, n/a, 300 K	(100)
47	D	1ben (human)	50 ns, SPC, 300 K	(101)
48	M	1xgl (human)	50 ns, SPC/E, 320 K	(102)
49	M	3inc (human)	10 ns, SPC, 300 K	(103)
50	M, D, H	4e7t (cow)	1.68 ns SA, IS, 383 K	(104)
51	M	3inc (human)	60 ns, TIP4P-IL, 300 K	(105)
52	A-chain	4ins (pig)	20 ns, TIP3P, 300 K	(106)
53	M	n/a (human)	100 ns, TIP3P, 310 K	(107)
54	H	1aiy (human)	600 ns, SPC/E, 300 K	(52)
55	M	2g4m (pig)	1.2 *µ*s, n/a, 300 K	(108)
56	M	1zni (pig)	300 ns WT-BEMD, TIP3P, 300 K	(109)
57	D	3w7y (human)	105 ns, SPC/E, 300 K	(110)
58	H	1mso (human)	1.6 *µ*s, SPC/E, 300 K	(111)
59	A-chain	4ins (pig)au	20 ns aMD, TIP3P, 300 K	(112)
60	D	4ins (pig)	MM-GBSA/TI, TIP3P, 300 K	(113)
61	D, H	5mt9, 1os3 (human)	2 *µ*s, SPC/E, 300 K	(114)
62	D	3w7y (human)	180 ns PTMetaD-WTE, TIP3P, 290 K	(115)
63	DPI, M	3e7y (human)	REMD, TIP3P, 340 K	(116)
64	H	3w7y (human)	WT-MD, SPC/E, 300 K	(117)
65	D	4ins (pig)	TBS, TIP3P, n/a	(118)
66	D	3w7y (human)	PTMetaD-WTE, TIP3P, 340 K	(119)
67	M	3inc (human)	100 ns, TIP4P-IL, 298 K	(120)
68	M	5ena (human)	100 ns, SPC, 300 K	(121)
69	M	2bn3 (cow)	50 ns, TIP3P, 300 K	(122)
70	D	4ins (pig)	MM-PBSA/SMD/US, TIP3P, 298 K	(123)
71	D	3w7y (human)	1 *µ*s/metadynamics, SPC/E, 300 K	(124)
72	H	1ai0 (human)	1 *µ*s, TIP3P, n/a	(125)
73	M	2m1d (human)	350 ns/100 ns REMD, TIP3P, 400 K	(126)
74	D	3w7y (human)	String method/ABMD/REUS, TIP3P, 303.15 K	(127)
75	M	1zeh (human)	100 ns, IL-TIP4P, 303 K	(128)
76	D	3w7y (human)	MSM, TIP3P, 310 K	(128)
77	M	2m1d (human)	500 ns, TIP3P, 300 K	(129)
78	D	3w7y (human)	PTMetaD, TIP3P, 290 K	(130)
79	D	3w7y (human)	REMD/metadynamics, n/a, 290-620 K	(131)
80	M	2jv1 (human)	Parallel tempering/MSM, TIP3P, 303-360 K	(132)
81	H	1znj (pig)	ABMD, TIP3P, 303.15 K	(133)

Shown in tabulated data are PDB codes (where known; n/a, not available) and simulation conditions including water models SPC, TIP3P, TIP4P, and their variants. In the column named “Simulation parameters”, n/a, not available is used for unknown details on simulation length, water model, and the temperature in a given simulation study. Moreover, in the same column, no details on simulation length are provided for those studies that used energy minimization or biased sampling techniques. Additionally, the following abbreviations are used: ABMD, Adiabatic-Bias Molecular Dynamics; aMD, accelerated molecular dynamics; BE-META, bias exchange metadynamics; CMEPS, computational mutations to estimate protein stability; D, insulin dimer; DPI, despentapeptide-(B26-B30)-insulin; DASI, diaminosuberoyl insulin (A1-B29 crosslinked); EM, energy minimization; H, insulin hexamer; IL, ionic liquid; IS, implicit solvent; M, insulin monomer; MM-GBSA, molecular mechanics-generalized Born surface area; MM-PBSA, molecular mechanics-Poisson Boltzmann surface area; MSM, Markov state model; ProI, proinsulin; PTMetaD, parallel-tempering metadynamics; PTMetaD-WTE, parallel tempering metadynamics in a well-tempered ensemble; RAMD, random acceleration molecular dynamics; REMD, replica exchange molecular dynamics; REUS, Replica Exchange Umbrella Sampling; SA, simulated annealing; SMD, steered molecular dynamics; TEM, targeted energy minimization; TBS, tempered binding simulation; TMD, targeted molecular dynamics; TI, thermodynamic integration; US, umbrella sampling; PEG-M, Pegylated insulin monomer; WT-BEMD, well-tempered bias exchange metadynamics; WT-MD, well-tempered metadynamics.

### 2.1 1985-1990

Over time, a substantial effort has been invested by the computational research community in unraveling the underlying complex mechanisms of biomolecular structures and their interactions using MD simulations, among which insulin has played the role of a key model protein. A molecular simulation was first performed on insulin in 1985 by Wodak et al. (56) to understand the conformational changes in the monomeric, dimeric, and hexameric forms using convergent energy minimization. This study mainly focused on understanding the conformational deviations between the monomer 1 and the monomer 2 in the insulin dimer. The minimization was done in the absence of solvent and with a modified all-atom CHARMM force field (ff) (135) where a marginal contraction in the protein volume was observed. The study noted atomic fluctuations within insulin and also validated the ff with explicit aliphatic hydrogens. As insulin was structurally a well-studied protein, it was also selected among several small molecules, nucleobases, and small proteins during the development and validation of all-atom molecular mechanics force fields (57). Krüger et al. (58) applied a larger van der Waals radii to all atoms (144) to avoid the protein contraction *in vacuo* and performed an independent 120 ps MD simulation of each monomer of the insulin dimer with the GROMOS ff (145). The simulation study probed modest conformational variations between monomers 1 and 2. Though the simulation studies of full insulin in an aqueous medium were not reported before 1990s, the despentapeptide insulin (DPI, insulin without the B26-B30 residues) analog was simulated in explicit solvent and counter ions (59). The simulation of the crystalline DPI with 4 molecules of DPI, 398 SPC water molecules (146), 4 Cd^2+^, 8 Na^+^, and 4 Cl^−^ ions was performed for 31 ps using the GROMOS ff. The positional fluctuations of the C_*α*_ atoms from this MD simulation were consistent with the B-factors obtained from the crystal diffraction data.

During this time period, simulations were mostly performed in vacuum using united-atom (GROMOS) and all-atom (CHARMM) force fields to study the conformational stability of insulin (monomer, dimer, and hexamer). These studies included energy minimization and short MD simulations (≤ 120 ps) to compare the structural rigidity between individual monomers in the insulin dimer, and residue flexibility between the A-chain and the B-chain in the insulin monomer. These early studies laid the foundation for future studies on insulin using MD simulations.

### 2.2 1991-1995

Several independent theoretical studies and a few experimental studies in conjunction with the MD simulations were published within this era. Most importantly, simulations of each insulin monomer from the 2Zn insulin dimer in aqueous solution and with counter ions were conducted for the first time on a 100 ps time scale (62). They also conducted an explicit water simulation of the insulin dimer to demonstrate key inter-residue interactions between each monomer involved in the dimerization. This study revealed that both insulin monomers exhibit significant conformational flexibilities in an aqueous medium, while monomer 1 was comparatively more stable than monomer 2. A detailed analysis was also performed on simulation trajectories of eight different conformers of insulin (5 monomers and 3 dimers) *in vacuo* using the CHARMM ff (60). The observed structural changes, salt bridging interactions, hydrogen bonding patterns, and the overall helical geometry provided a detailed perspective on the conformational dynamics of insulin. These findings were suggested to be in good agreement with the experimental studies (147–150).

Other set of studies introduced targeted energy minimization (TEM) and targeted molecular dynamics (TMD) to probe the conformational transitioning between the T↔R states of the insulin monomer (63, 64, 67). A geometrical constraint (along dihedral angles *ϕ* and *ψ*) was applied to facilitate the conformational change along a pre-defined pathway from the starting conformer (T or R) to the known target conformer (R or T). The stable intermediates and the energy barriers along the transitioning of the B1-B8 residues from an extended conformation in the T state to a helical conformation in the R state, and *vice versa*, were explored in atomic detail. Tidor and Karplus (66) estimated the change in the free energy of association of insulin monomers into a dimer as –7.2 kcal/mol which is in agreement with the experimental dimerization free energy (ΔG_dimerization_) obtained using concentration difference spectroscopy (151).

Several insulin derivatives or analogs were also reported, among them the single-chain insulin with a crosslink between the A1 and B29 residues was well-studied experimentally (152–155). The reported single-chain insulin was biologically inactive (156, 157). To explain the inactivity of the crosslinked insulin, Krüger et al. (65) simulated the single-chain insulin for 100 ps in explicit water and concluded that the C-terminus of the B-chain is more rigid and some of the residues that are significant for interaction with the receptor are shielded in the bridged insulin which hinders its binding to IR. We also observed that a few research groups implemented both experimental (X-ray and NMR) and simulation techniques to resolve the structure and dynamics of insulin or surrounding water molecules (46, 61). For example, Badger and Gaspar (61) constrained the insulin atoms during MD simulations to determine the configurations of water molecules within the cubic insulin crystal lattice. The study reported that the network of water molecules near the hydrophilic surface of the insulin switches between pentameric and hexameric configurations. Knegtel et al. (46) performed energy-minimizations and restrained MD simulations *in vacuo* to inspect the conformational flexibility of the DPI protein. This study concluded that the residues GlyA1, AsnA21, and PheB25 were more flexible. NMR studies performed in their work suggested that DPI adapts a conformation similar to molecule 1 in the insulin dimer but the conformer derived after restrained MD simulations resembles molecule 2. The anomalies between the NMR and MD observations were attributed to a shorter simulation time and limitations in the force-fields.

During this time period, we noticed that insulin was simulated in near physiological conditions: aqueous environment with counter ions. The studies used more refined force fields to elucidate the inter and intra insulin interactions and studied the role of water in the conformational flexibility of insulin with microscopic details. The thermodynamic parameters during the insulin dimerization were also estimated using all-atom MD simulations. Other than MD simulations, targeted methods (TEM and TMD) were applied to explain the conformational transition of insulin between the T and R states, or *vice versa*. We also observed that the simulations of insulin analogs (DPI and single-chain biologically inactive insulin) were performed to provide possible explanations for the experimental observations. In brief, we found that advanced simulation techniques were applied to complement experimental studies on the insulin structure during this period.

### 2.3 1996-2000

The pathways of conformational transitions between the T_6_ ↔T_3_ R_3_↔R_6_ states of the insulin hexamer were further probed using targeted (TEM and TMD) methods with explicit-solvent simulations on a 100 ps time-scale and with the GROMOS ff (68). The potential energy of the transient conformers and the cooperative transitioning of the N-terminal region (B1-B8 residues) of the B-chain were observed in detail. Another study incorporated restrained MD simulations and simulated annealing along with the NMR spectroscopy to predict and refine the phenol-stabilized R_6_ conformer of the insulin hexamer (69).

At the same time, a relatively long MD simulation (1000 ps, i.e., 1 ns) of the insulin monomer was performed at higher pressures (varying between 1 bar and 20 kbar) to unfold the protein and compare its stability with the ribonuclease A protein (71). This study reported that insulin maintains its structural stability above 13.2 kbar, while ribonuclease A denatured even at a lower pressure of 8.5 kbar. It was also observed that a network of hydrogen bonds formed by water molecules around each protein played a crucial role in maintaining their stabilities. A 5-membered ring structure of water molecules was dominant in ribonuclease A, whereas a 6-membered ring structure of water molecules around insulin was observed to stabilize it at higher pressures. Moreover, this study also demonstrated that the *α*-helix is relatively more stable than the *β*-sheet, which contributed to a higher stability of insulin which only has *α*-helices compared to ribonuclease A which has both types of secondary structural components.

Cheng and Rossky (70) performed MD simulations in aqueous media and estimated the hydration thermodynamics of polar and charged residues at the dimeric interface to probe the significance of the structure and energetics of the B-chain in 2ZN insulin. This study also reported the orientation and the entropy of water molecules in the vicinity of polar and charged residues, and hydrophobic surfaces influencing the dimerization of insulin.

To summarize this period, we found that the simulation time was increased from ps to ns time-scales. The conformational stability of insulin in drastic conditions (e.g., a higher pressure) was also probed using MD simulations which is non-trivial to explain using experimental methods alone.

### 2.4 2001-2005

Falconi et al. (72) performed circular dichroism (CD), fluorescence spectroscopy, two-dimensional NMR, and all-atom MD simulations of the insulin dimer for 600 ps to probe the interactions of a few monosaccharides with the hormone. These MD simulations suggested that D-glucose prefers to dock in the binding pocket formed by the side-chains of residues TyrA14, ValB2, and LeuB17 in the insulin dimer surface. The glucose binding surface inferred from MD simulations was reported to agree well with the Nuclear Overhauser Enhancement (NOE) method in the NMR spectroscopy. In another study, Falconi et al. (73) thoroughly investigated the rigidities of the monomers in the insulin dimer using atomistic MD simulations and inferred that monomer 1 exhibits a higher inflexibility than monomer 2. They estimated the contribution to the free energy of dimer formation for each residue of the insulin monomer involved in the formation of the insulin dimer. Among the B-chain residues, Gly8, Glu13, Gly23, and Thr27 exhibited lower contributions to insulin dimerization; Ser9, Phe24, Tyr26, and Pro28 exhibited a medium contribution; and Phe25, Val12, and Tyr16 exhibited a higher contribution to the dimerization process (73). The key residues of insulin crucial for the dimerization inferred from this simulation study corroborated well with the experimental mutagenesis studies (158, 159). Karplus and coworkers also used molecular mechanics–generalized Born surface area (MM-GBSA) method to estimate the binding free energy (–11.9 kcal/mol) of dimerization of insulin and delineated the key residues in the dimer interface (80). In another study, Zoete et al. (80) investigated using docking and MD simulations the putative binding sites for D-glucose on the insulin surface. Their study showed that glucose preferably interacts with the residues ValB2 and LeuB17 with a binding affinity between –1.4 and –3.5 kcal/mol. They also discussed the stability and conformational dynamics of the insulin monomer and the dimer in microscopic detail using longer simulation (~5-10 ns) (77). MD studies also confirmed that the R_6_ insulin hexamer is more stable in the presence of Zn^2+^ ions and phenol (74, 75). The electrostatic interactions and hydrogen bonds appeared crucial for maintaining the stability of the hexamer (74).

Swegat et al. (75) applied a constrained MD simulation approach to track the dissociation pathway of a phenol molecule from its binding site on the R_6_ insulin hexamer and estimated the free-energy profile along the distance between the center of mass of phenol and the insulin hexamer as a reaction coordinate. This study explained the structural events within insulin during phenol dissociation. The benzene ring of the phenol molecule noticeably reoriented and approached the exit channel between the residues HisB5 and CysA11. The side chain of IleA10 was noted as a major hindrance for phenol escape. The study also calculated the free energy of dissociation of phenol from the R_6_ insulin hexamer as 21.8 ± 2.8 kJ/mol.

Budi et al. (78, 79) used MD simulations to study the conformational stability of insulin or its B-chain using chemical stress, thermal stress, and electric field. All disulfide bonds in insulin were reduced and the temperature was elevated to 400 K during these simulations to emulate the chemical and thermal stress, respectively (78). The insulin molecules with the native disulfide bonds at 300 K and 400 K were more rigid than the ones without the disulfide bonds at respective temperatures. Notably, the B-chain behaved similarly among insulin molecules with or without disulfide bonds at ambient temperature, thereby implying that the B-chain of insulin folds independently. This observation is in line with the reported experimental findings (160, 161). The authors also applied static and oscillating electric fields, ranging from 10^7^ to 10^9^ V/m to study the stability of the B-chain of insulin using MD simulations (79). The conformational states explored by the B-chain suggest that the secondary structure of the peptide was fully disrupted in an oscillating electric field, in comparison to the partial melting observed in a static electric field. This MD study, although not physiologically relevant, provided insights into the stability of a peptide under the influence of an electric field, which is a challenging task to investigate experimentally.

During this time period, the simulation length and the constituents in the simulation domain were increased with better computational facilities and MD simulation codes. Earlier studies inferred that molecule 1 exhibits higher structural stability than molecule 2 in the insulin dimer, but the contributions of key interface-residues to the stability of the insulin dimer were probed in detail during this time period. The mechanisms of interactions of glucose and phenol with insulin were also studied for the first time. The stability of the B-chain of insulin under chemical stress, thermal stress, and electric fields were also investigated using MD simulations.

### 2.5 2006-2010

In a comprehensive study, Zoete and Meuwly (82) assessed the effect of Ala-mutations (in a few crucial residues LeuA16, TyrA19, LeuB11, LeuB15, ArgB22, and PheB24) on the stability of insulin. They implemented computational mutations to estimate the protein stability, and molecular mechanics generalized born surface area (MM-GBSA) methods to calculate the relative change in the free energy due to each mutation. They found that mutating aforementioned selected residues leads to misfolding of insulin and probably hinder its binding to the receptor. Haas et al. (90) studied the effect of change in pH on the structure of the insulin monomer and estimated the conformational entropy using MD simulations. A correlation between the underlying structural changes due to the pH variation and aggregation of insulin was reported. Yarovsky and coworkers applied several MD simulation methods to broadly explore the stability, dynamics, folding, and aggregation of insulin or its B-chain in different simulation conditions (83). The results showed that both termini of the B-chain were highly flexible and the B-chain chiefly adopts the T state conformer. The B-chain of insulin was also subjected to simulations in varying temperatures (300 K and 400 K) to understand the packing interactions and conformational transitions within the protein. The stability of the B-chain of insulin was also monitored under thermal stress and electric fields of varying forms, frequencies, and strengths (84, 85). As expected, the disulfide bonds stabilized the overall structure of the B-chain in comparison to the isolated B-chain. This observation is in line with a previous study reported by Budi et al. (78) which states that the isolated B-chain destabilizes under thermal stress. They also systematically compared the conformational evolution of the B-chain of insulin in different force fields (CHARMM27, OPLS, AMBER03, GROMOS 43A1, and GROMOS 53A6) (86). Among these force fields, the dynamic behavior of insulin elucidated from the CHARMM and the GROMOS 43A1 trajectories on a 50 ns time-scale agreed well with the experimental observations.

In another work, Yarovsky’s group first implemented bias exchange metadynamics (BE-META) technique on the B-chain of insulin to investigate the intermediate states along the folding pathway and estimated the respective free energies (88). The extended conformation of the B-chain was subjected to independent metadynamics simulations along with seven distinct collective variables (CVs) and replicas were allowed to exchange between them at regular frequencies. In this study, the free energy wells in the energy landscape were successively filled by small Gaussian potentials which allow the system to overcome underlying free energy minima (162, 163). They were able to characterize three distinctly populated basins in the free energy. The first basin was populated by a molten-globule structure governed by electrostatic interactions, the second basin represented a molten-globule structure governed by hydrophobic contacts, and the third basin resembled the near biological folded conformer of the B-chain of insulin (92). The transition from the second molten-globule state to a near native conformer was required to cross a higher energy barrier during BE-META (88).

**Figure 4:**
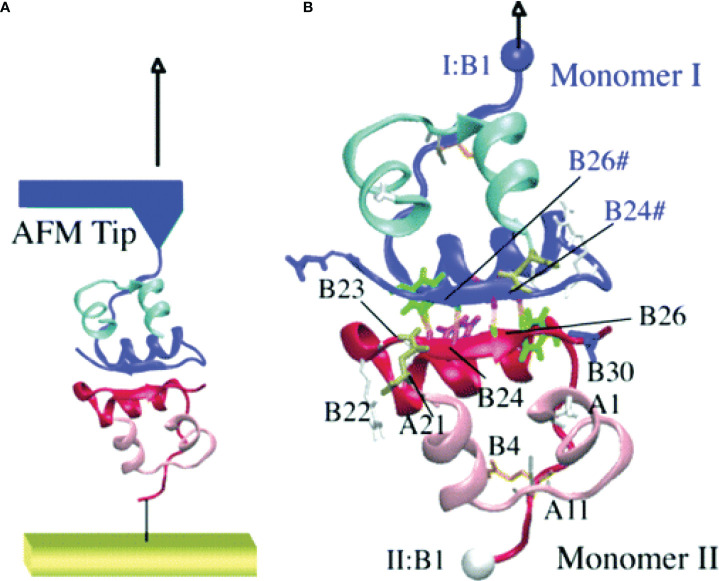
Steered molecular dynamics (SMD) simulations of the insulin dimer. **(A)** A schematic model of the AFM experiment with the insulin dimer as the target protein. **(B)** The C*α* atom of PheB1 of monomer I (top) is pulled after attaching a harmonic spring potential, while the C_*α*_ atom of PheB1 of monomer II (bottom) was held fixed. The residues involved in monomer-monomer interactions are labeled. Reprinted with permission from Kim et al. (81), Copyright 2006 American Chemical Society.

The steered molecular dynamics (SMD) technique was also employed to mimic the observations from the atomic force microscopy (AFM) experiments to understand the unfolding of the insulin dimer (81). The C_*α*_ atom of PheB1 of a monomer in the insulin dimer was kept rigid and the same atom of the other monomer in the dimer was pulled with different velocities ranging between 0.0025 and 0.01 nm/ps during the force-induced dissociation of the insulin dimer (**Figure 4**). This study demonstrated that the dissociation of insulin is a rate- and pathway-dependent process, and the unfolding events of insulin agree qualitatively with the AFM experiment. Vashisth and Abrams (87) performed random acceleration MD (RAMD) and multiple SMD simulations in conjunction with the Jarzynski’s equality to identify the escape pathways of phenol and to estimate the unbinding free energy of phenol from the R_6_ insulin hexamer. The study reported three probable escape paths for phenol and characterized the disruption of interactions between phenol and the residues in the insulin hexamer in atomic detail. The change in the free energy of escape estimated for each pathway (18.06 k_B_T, 17.75 k_B_T, and 20.60 k_B_T) was obtained from the second-order cumulant expansion of Jarzynski’s equality. Previously, Swegat et al. (75) had studied the dissociation pathway and energetics of phenol dissociation from the R_6_ insulin hexamer. However, they selected a single reaction coordinate and reported only a single phenol dissociation pathway. The study by Vashisth and Abrams outlined multiple competing dissociation pathways and provided additional details during phenol dissociation including the role of water molecules near the phenol binding pocket.

To probe the adsorption dynamics and inherent structural changes in insulin or its B-chain on graphene surfaces or on single-walled carbon nanotubes (SWNTs), a research group from China carried out explicit water MD simulations (89, 91). They tested the adsorption of insulin on graphene surfaces of different sizes, shapes, and flexibilities. Insulin was reported to interact with the non-polar, as well as, polar and charged residues on the graphene surfaces. The π-π stacking between the phenyl rings and the graphene surface contributed significantly to the adsorption of insulin. However, insulin lost its native fold after absorption on the graphene sheet within a short simulation period (10 ns) (91). Short MD simulations (6 ns) of the B-chain of insulin performed on different charged SWNTs suggested that the electrostatic interactions were dominant during the adsorption of the peptide on charged SWNTs than on uncharged SWNTs (89). The ordered first hydration shell around the charged SWNTs also promoted the adsorption of the peptide. The secondary structure of the B-chain during adsorption on SWNTs was more preserved than the secondary structure of insulin on graphene sheets. The understanding from these studies envisaged the application of insulin in the fields of biosensors, biomaterials, biomedical devices, and drug delivery.

The effect of residue mutations, pH, thermal stress, chemical stress, and external pulling force on the stability of the insulin structure was probed using different simulation techniques during this time period. The folding pathway of the B-chain of insulin was investigated using the metadynamics method (164), which has previously not been applied. The stability of insulin on graphene surfaces or SWNTs and the mechanism of insulin aggregation were also probed for the first time. Also, multiple escape pathways of phenol from the R_6_ insulin hexamer and their associated energy barriers were reported in detail.

### 2.6 2011-2015

Between 1985 and 2010, we found that the simulations of insulin were primarily focused on inspecting its conformational dynamics and stability using short MD simulations (mostly ≤ 10 ns). From 2011 onward, we will find that relatively longer simulations (10-100 ns) were performed to probe various aspects of the insulin structure. Yang et al. (93) conjugated insulin with the polyethylene-glycol (PEG) polymers of different chain lengths to investigate their impact on the structure and the stability of insulin using all-atom MD simulations. The PEG polymer entangled around insulin during these simulations and resulted in a larger volume of the PEGylated insulin and a lesser solvent accessible surface area of insulin. The conjugated insulin maintained its secondary structure at an elevated temperature of 450 K. The study provides atomistic insights into the role of the PEG on the stability and bioavailability of conjugated insulin, which may inspire future work in the development of PEGylated insulin therapeutics.

Bagchi and Roy performed a detailed inspection of the interfacial waters near the dimer forming surface (DFS) and the hexamer forming surface (HFS) using MD simulations (97). They observed that the structural relaxation of hydrogen bonds established between the DFS and the interfacial water molecules is faster than the hydrogen bonds established between the HFS and the interfacial water molecules. Structured interfacial water near the HFS exhibits a higher residence time than those in the vicinity of the DFS which was attributed to a relatively slow decay in rotational auto-correlation function of the hydrogen bond vector around the HFS. The water molecules mostly rearranged to form clathrate-like structures with the H-atoms of water molecules directed outward near the largely polar solvent accessible area of HFS, but water molecules in the vicinity of DFS likely prefer the H-atoms directed inward. The study inspected the dynamic role of water molecules in forming the dimeric and hexameric states of insulin.

Insulin also tends to aggregate into amyloid fibrils, although the underlying mechanism for the formation of ordered aggregates remains elusive. Chinisaz et al. (96) conducted five sets of 15 ns long MD simulations in different types of salt (KCl and NaCl), temperature (345 K and 500 K), and acidic pH (of 2) to probe the structural changes within insulin and a truncated form of insulin (DPI) during the amyloid formation. In accordance with experimental observations, insulin initially unfolded and helical content decreased, followed by the formation of the *β*-sheet secondary structure. The salt concentration proliferated the *β*-sheet structure and DPI exhibited a higher instability compared to the full-length insulin at elevated temperatures. This simulation study also concluded that higher temperature, KCl salt, and acidic pH facilitate aggregation of insulin into an amyloid form. In another study by Mishra et al. (99), authors reported a peptide inhibitor that impedes the fibrillation of insulin. The experimental and the MD simulation results demonstrate that the peptide interacts with the A-chain helices and preferably with the FFY motif (B24-B26) of the B-chain, thereby preventing these key residues from establishing stable intra- and intermolecular interactions during fibrillation of insulin. Kim et al. (98) performed atomistic MD simulations to scrutinize the effect of mutating the CysA7 residue by Tyr on the stability of proinsulin. The mutation disrupted the α-helical propensity of the first helix in the A-chain and resulted in a partially folded intermediate with a flexible N-terminal region. This study suggested that the structural changes in the A-chain due to the mutation may facilitate the aggregation of proinsulin.

An attempt using MD simulation was also made to search for a chemical compound that can assist in the oral delivery of insulin (101). The *β*-cyclodextrin (*β*-CD), a cyclic glucose oligosaccharide consisting of seven glucopyranose units that are linked by *α*-(1,4) glycosidic bonds, was docked to insulin to study its interactions using MD simulations. Insulin was shown to accommodate a maximum of four *β*-CD molecules, but a complex of three *β*-CD with insulin was most favorable. MD simulations of 1:3 insulin–*β*-CD complex in explicit water showed that the complex is very stable on the 50-ns time-scale. Compared to the unbound insulin, the flexibilities in residues and the secondary structure changes in insulin decreased on formation of the insulin–*β*-CD complex. The understanding from this theoretical study may potentially motivate future work to develop oral insulin formulations.

**Figure 5:**
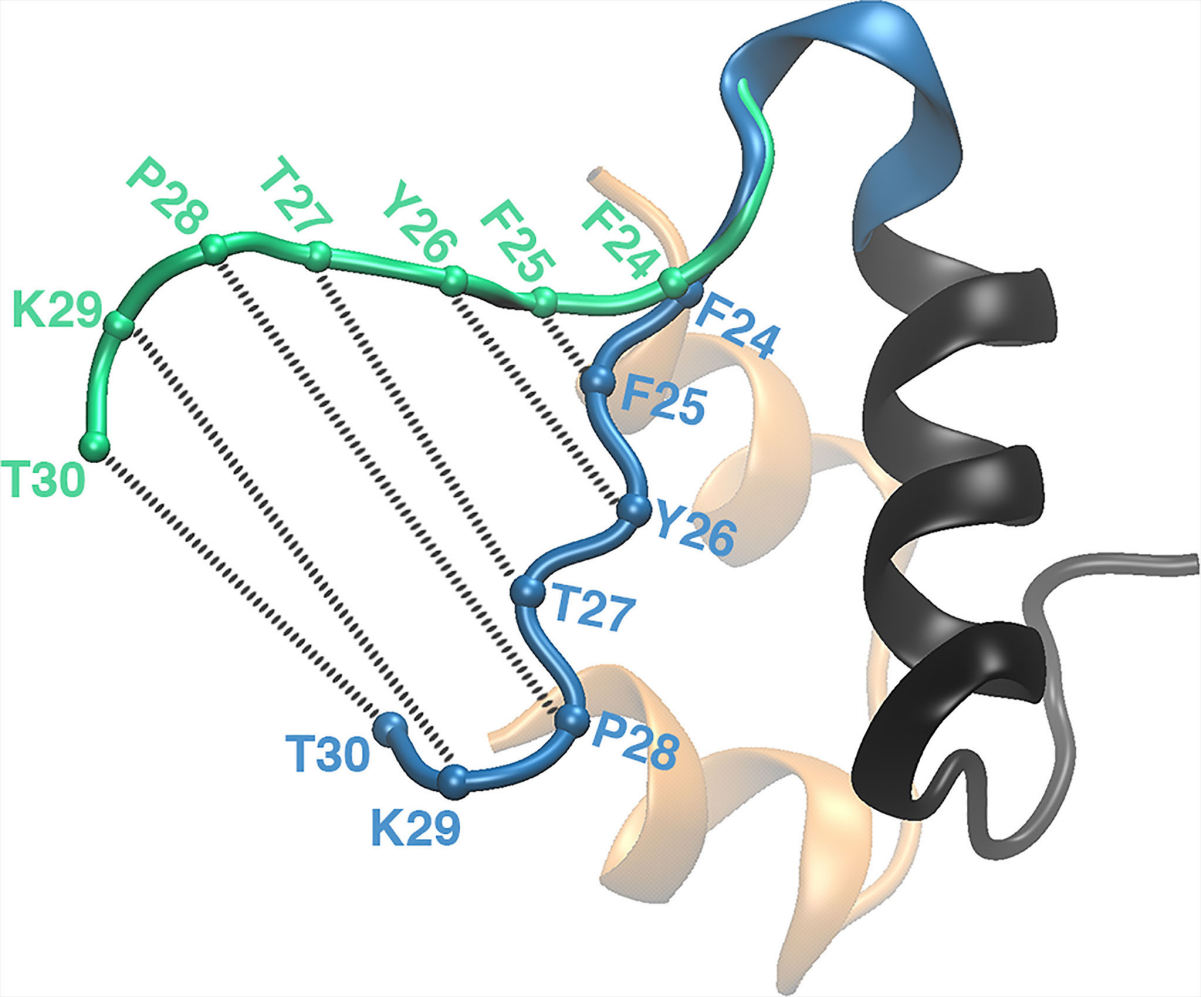
The zipper-like opening of the BC-CT with a hinge at F24. The BC-CT in its closed (inactive) and wide-open (active) conformations is shown in blue and green cartoon representations, respectively. The C_*α*_ atoms of the corresponding residues are represented by colored spheres. Reprinted from Papaioannou et al. (100), an open access article distributed under the terms of the Creative Commons Attribution License (http://creativecommons.org/licenses/by/4.0/).

Explicit solvent MD simulations, individually or in conjunction with experiments, were routinely used to understand the structural flexibility due to residue mutations and to establish the significance of the C-terminal residues (B24-B30) of insulin in the dimerization and receptor binding (94, 95, 100). Also multiple MD simulations (~120 ns long) were performed to elucidate the conformational changes in insulin on binding to IR (98, 100). They demonstrated that the B-chain C-terminal (BC-CT) residues (GlyB20-ThrB30) act as a zipper with a hinge at the PheB24 residue. The BC-CT remains closed in the inactive state, but it adopts a wide-open conformation to interact with the receptor (**Figure 5**). The hydrophobic interactions are more dominant in the closed inactive state and the residue TyrB26 plays a key role in retaining the hydrophobic core. Thus it was hypothesized that an insulin analog with the TyrB26Asn mutation will disrupt the hydrophobic core and favor the open active state of insulin.

The water dynamics around insulin and near the vicinity of the dimeric and hexameric interfaces of insulin were elucidated comprehensively during this period. Moreover, MD simulations were performed to understand the typical conditions that can facilitate the ordered aggregates of insulin into amyloid fibrils. This knowledge instigates research groups to propose peptide inhibitors or mutations in the A-chain of insulin that can regulate the aggregation of insulin. The significance of the zipper-like opening mechanism of the C-terminus of the B-chain in the activity of insulin was elaborated. The key residues in these flexible regions of the B-chain crucial for the insulin activity were delineated and potential mutations enhancing the insulin activity to IR were proposed. Simulations also suggested a cyclic glucose oligosaccharide formulation to facilitate the oral delivery of insulin and circumvent the adverse effects at the injection site.

### 2.7 2016-2020

In this time period, we found a significant increase in research studies (a total 27 studies over a 5-year period) on the application of MD simulation methods in insulin research. Baheri and Dayer (102) probed the effect of temperature and pH variation on the misfolding and aggregation of insulin. Insulin was observed to exhibit higher structural alterations at a low temperature and acidic pH conditions which was attributed to the misfolding and aggregation of the protein. Sklepari et al. (104) studied the effect of temperature on the structures of the insulin monomer, dimer, and hexamer using simulated annealing MD simulations to complement the observations from dynamic light scattering (DLS), CD, and NMR experiments. A higher temperature induces higher flexibility in the B-chain C-terminus, and to the N-termini of both A- and B-chains of the insulin monomer. The insulin dimer tends to dissociate at a higher temperature and the N-termini of both A- and B-chains exhibited higher flexibilities. Similarly, the insulin hexamer gradually inflates and dissociates at higher temperatures. This study also showed that at higher temperatures the *α*-helical content is more preserved in the insulin hexamer, followed by in the dimer, and least in the monomer.

**Figure 6:**
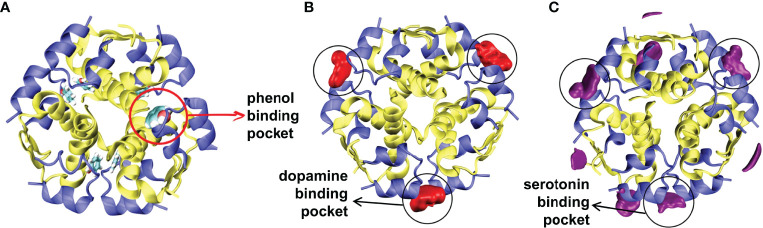
Spatial distributions of neurotransmitters. **(A)** The starting conformation of the R_6_ insulin hexamer with the phenol binding pocket is shown. Spatial distributions of **(B)** dopamine, and **(C)** serotonin around the insulin are depicted. Reprinted from Palivec et al. (52), an article published by Elsevier under the terms of the Creative Commons Attribution License (http://creativecommons.org/licenses/by/4.0/).

Pan et al. (105) investigated the effect of green solvents, imidazolium ionic liquids (ILs) with different alkyl chain lengths, on the structural stability of insulin using MD simulations. Interestingly, they observed that cations and ILs with shorter alkyl chains induce more stability to the insulin structure. In another study, Li et al. (120) conducted MD simulations and differential scanning calorimetry (DSC) experiments to characterize the conformational stability of insulin in 17 IL solvents. The results illustrate that insulin preserves its native structural components in pure ILs with a shorter alkyl chain and lower hydrogen bond basicity. Soleymani et al. (103) compared the binding interactions and the stability of the insulin dimer in the presence of vitamin D_3_ and vitamin E using spectroscopy, DSC, and MD simulation. The study showed that insulin loses its secondary structure content in the presence of vitamin D_3_, but vitamin E stabilizes the native conformation of insulin. Another study illustrated that vitamin E perturbs the B-chain C-terminal residues and adapts a wide-open/active conformational form of insulin (121). Palivec et al. (52) investigated the conformational stability of the insulin hexamer in serotonin, dopamine, and arginine solutions using MD simulations and protein crystallography. The study revealed that the neurotransmitters (dopamine and serotonin) bind and enhance the stability of the insulin hexamer (**Figure 6**). Among these small ligands, serotonin preferentially facilitates the transition of the R_6_ state to the T_3_R_3_ state.

Atabay et al. (107) investigated the adsorption of insulin on biocompatible nanosheets (graphene monoxide, silicon carbide, and boron nitride) using MD simulations. The non-bonded interaction energy between insulin and nanosheets, key interacting residues, and the role of interfacial water molecules were probed in atomic detail. They concluded that insulin strongly interacts with the nanosheets by its flexible C- and N-termini. The probability density function and the potential of mean force calculations were performed to understand the effects of mutations on the dynamics of the BC-CT opening and the energetics of insulin activation (108). Several studies revealed that BC-CT unzips from the hydrophobic core to facilitate binding of insulin to IR (40, 95, 165–167). Thus a closed and wide-open conformer of BC-CT were suggested to resemble the inactive and active states of insulin, respectively. The study considered three hyperinsulinemia analogs of insulin, with PheB24Ser, PheB25Leu, and ValA3Leu mutations, and three more critical analogs after mutating the hinge residue TyrB26 to Ala, Glu, and Ser. The hyperinsulinemia analogs mostly exhibited the closed BC-CT conformer corroborating their reduced activity. On the other hand, critical analogs exhibited a higher probability to attain the wide-open conformer of BC-CT, which implies their enhanced activity for IR in comparison to the WT-insulin. Specifically, the MD study showed that insulin analogs with TyrB26Glu and TyrB26Ser mutations exhibited significant enhanced activity.

Singh et al. (109) used well-tempered bias exchange metadynamics simulations to characterize the equilibrium ensembles of insulin at low pH and high temperature. The study demonstrated that insulin adapts two low-energy metastable intermediate states. These folding metastable conformers of insulin exhibited a native-like topology with 63% native hydrophobic contacts, and corroborated well with the experimental data obtained from spectroscopic, crystallographic, and calorimetric measurements during early stages of insulin aggregation. Duboué-Dijon et al. (111) performed MD simulations of insulin with different types of salts (NaCl, CaCl_2_, and MgCl_2_) to provide a detailed atomistic view of ion-protein interactions. They also found that the standard full charge force field (168) overestimates the binding of Ca^2+^ ions to carboxylate groups in comparison to the Electronic Continuum Correction (ECC) force field (169). Brezina et al. (114) investigated the effect of arginine on the stability of the insulin dimer and hexamer at low and high ion concentrations using crystallography, MD simulation, and capillary electrophoresis techniques. At high ionic concentration, arginine docked around the shallow binding site formed by the B1-B8 loop of the insulin dimer and established interactions with the HisB5, GluB13, and HisB10 residues. The binding of arginine stabilized the insulin dimer and hindered the transition to a hexameric state. Santra and Jana (126) reported a detailed study on the effect of arginine solution of varying concentrations (0.5M, 1M, and 2M) on the stability of the insulin monomer at near-physiological (300 K) and elevated (400 K) temperatures using atomistic MD and replica exchange MD (REMD) simulations. They revealed that the insulin monomer unfolds completely at 400 K in an aqueous medium, while in a 2M arginine solution the hormone maintains its native fold. The study revealed microscopic details of favorable interactions between the carboxyl groups of arginine and the aromatic amino acid residues of insulin.

The underlying role of water molecules on the stability of the insulin dimer and hexamer was investigated in detail during this time period. Mukherjee et al. (110) probed the significance of cavity water in stabilizing the hexameric state of insulin using MD simulations, X-ray crystallography, and quantum calculations. The confined water molecules formed a stable octahedral geometry with three HisB10 residues per Zn^2+^ ion. The water molecules also established a strong hydrogen-bond network in the core of the insulin hexamer. The hexamer architecture of insulin was significantly perturbed in the absence of these confined water molecules. Raghunathan et al. (113) estimated the relative stabilization free energy of mutated insulin dimer analogs (by mutating residue PheB24 to Ala and Gly) relative to that of the wild type dimer using the thermodynamic integration method. The inter-monomer hydrogen bonds were destabilized and water-mediated hydrogen bonds were more prominent in the mutated insulin dimer, which disrupted the anti-parallel *β*-sheet formed by the C-terminus of the B-chain at the monomer-monomer interface, thereby decreasing the stability of the insulin dimer.

Banerjee et al. (115) probed the energetics and the role of water molecules in dissociation of the insulin dimer using parallel tempering metadynamics simulations in a well-tempered ensemble (PTMetaD-WTE). They demonstrated the intermediates along the dissociation pathway of the insulin dimer. The dynamics of water molecules altered significantly along the dissociation pathway and the density of water increased notably around residues PheB24, PheB25, and TyrB26 (which are also involved in the anti-parallel *β*-sheet of the insulin dimer), which further facilitated dissociation of the insulin dimer. In another study (124), Banerjee and Bagchi performed biased metadynamics (PTMetaD-WTE) and long unbiased (~1-2 µs) simulations to augment the role of water molecules during insulin dimerization and dissociation process. Notably, the minimum energy path of dimer dissociation calculated from biased metadynamics simulation was comparable to the dimer association path calculated from unbiased MD simulations. The study also revealed that the water molecules possess faster rotational motion at a higher separation (~5 nm) between the monomers in the insulin dimer. However, the number of confined water molecules and their dynamics significantly diminished at a lesser separation (~2 nm). It was suggested that the proper orientation of residues in the hydrophobic hotspot region (formed by residues PheB24, PheB25, and TyrB26) in each monomer leads to dewetting and facilitates the dimerization of insulin. Yusuff et al. (125) conducted MD simulations of insulin hexamers with and without Zn^2+^ ions in the hexamer’s cavity. The study unambiguously showed that the insulin hexamer is fairly rigid during MD simulations in the presence of Zn^2+^ ions. However, without Zn^2+^ ions the insulin residues exhibited higher fluctuations and the interatomic distances between the six GluB13 residues increased leading to a partial destabilization of the structure.

Shaw and co-workers applied “tempered binding” and long-timescale conventional MD simulations to investigate the events during the dimerization of five structurally and functionally diverse protein–protein systems, including insulin (118). The kinetics and the association rates in the protein–protein complexes were evaluated at the molecular level. The newly introduced “tempered binding” metadynamics method dynamically scales the strength of interatomic interactions and impedes conformational trapping due to improved sampling. Gong et al. (123) estimated the absolute binding free energy of insulin monomers into a dimer using the SMD method and the confinement method based on a nonphysical thermodynamic cycle. The estimated binding free energy, –8.97 ± 1.41 kcal/mol, was consistent with the reported experimental value, –7.2 ± 0.8 kcal/mol (151). The study also revealed that ValB12, TyrB16, PheB24, PheB25, and TyrB26 are crucial residues for the dimerization of insulin. Antoszewski et al. (127) also applied multiple advanced MD simulation methods including SMD, the string method, adiabatic-bias MD, and the REMD to explore in detail the dissociation pathways, energetics, and subtle conformational transitions in monomer during the dissociation of the insulin dimer. They observed multiple dissociation pathways of the insulin dimer and focussed on two critical dissociation/association pathways: *α* and *β* paths. Along the *α* pathway, the interfacial *α* helices in the B-chain initially move away and get solvated, and along *β* path the interfacial anti-parallel *β* strands in the B-chain first separate and get solvated. The unfolding intermediates of the insulin dimer along these two limiting paths were charaterized and noted to be distinct. The study reported that the *α* path is the energetically favorable dissociation/association track.

The significance of amphiphilic solvent ethanol on the stability of the insulin dimer, and the free energy surface during the dissociation was probed using biased MD simulations (119). Ethanol was observed to lower the free energy barrier for dissociation of the insulin dimer by diminishing the interactions between two antiparallel *β*-sheets and later stabilizing the insulin monomers. Ethanol also showed a tendency to replace water molecules from the insulin hexamer, thereby contributing to the destabilization of the insulin structure (117). Ethanol perturbed the symmetry of the insulin hexamer by expelling water molecules from the core cavity and establishing hydrogen bonding with the GluB13 residues and sometimes with the HisB10 residue.

The adsorption of the A-chain of insulin on bare and functionalized silica surfaces was scrutinized using accelerated MD simulations (106, 112). The electrostatic interactions and the dipole moment were shown to play a major role during the adsorption of the A-chain of insulin on the polar silica surface. However, vdW interactions led to the adsorption of the A-chain of insulin on a non-polar silica surface. The *α* -helix in the A-chain of insulin completely melted on a non-polar silica surface, although the secondary structure was partially stable on a polar surface (106). Nejad and Urbassek (112) also investigated the role of surface polarity in the adsorption of the A-chain of insulin on functionalized silica surfaces. Insulin adsorbed on hydroxylated and carboxylated surfaces with a lower affinity and exhibited subtle conformational changes. However, the peptide adsorbed firmly on a methylated silica surface and underwent complete denaturation of its secondary structure.

Hosseinzadeh et al. (116) explored the conformational dynamics of insulin with ZnO nanoparticles by REMD simulations. The polar and charged residues of insulin interacted with the ZnO nanoparticle, although insulin only marginally unfolded on the nanoparticle surface. The study demonstrated that the N-terminus of the A-chain and the N-terminus of the B-chain interact preferably with the nanoparticle and water molecules, respectively, which induce opposite directional movement of both chains, thereby causing the unfolding of the insulin structure. The insulin residues GlyA1, AsnA21, HisB10, GluB13, TyrB16, GluB21, and ArgB22 interacted preferably with the ZnO nanoparticle. The mechanistic details observed from this study agreed well with the previous experimental study (170). Kurpiewska et al. (122) considered insulin as a model protein and investigated the role of high pressure on the stability and aggregation of the protein using crystallography and MD simulations. The study reported that the B-chain of insulin is more susceptible to a higher pressure than the A-chain. The residues in both termini of the B-chain (B1, B2, B29, and B30) exhibited a higher flexibility at higher pressures. The authors anticipated that the termini of the B-chain probably contribute to the amyloidogenesis of insulin. They deposited the crystal structure of each insulin at different experimental pressures (PDB codes 6QQ7, 6QQG, 6QRH, and 6QRK).

The stability of insulin in various ILs and in the presence of arginine, vitamins (D_3_ and E), divalent cations (Ca^2+^ and Mg^2+^) and neurotransmitters (serotonin and dopamine) using MD simulations were major topics of study during this time period. The significance of the BC-CT of insulin in its activity was highlighted earlier using MD simulations and the mutations that can diminish or enhance the activity of insulin toward IR were suggested. The role of water molecules on the stability of insulin and in the dissociation of the dimer was probed by advanced sampling methods. Two critical dissociation pathways of the insulin dimer were reported, among them, the unfolding events of the insulin monomer along the *α* path were in agreement with the minimum free energy pathway reported by Banerjee et al. (115). The conformational stability of insulin over silica surfaces and ZnO nanoparticle was also studied.

### 2.8 2021-To Date

In this most recent period, MD studies are still being conducted using advanced simulation methods to elucidate the conformational dynamics of insulin, the role of water in the structural stability, and the thermodynamics of dissociation of insulin dimer primarily. Bagchi and coworkers have performed detailed studies of insulin to characterize the microscopic and mechanistic facets of the dissociation of the insulin dimer (130, 131, 171). The projection of the free energy landscape along different parameters revealed several metastable states sampled during insulin dimerization. The partial destabilization of the hydrogen bonds (HBs) between the anti-parallel *β*-sheets, followed by stabilization of the partially unzipped dimer by water, and finally a complete loss of HBs led to the entry of more water molecules in the intermonomeric junction leading to dissociation of the insulin dimer (130). They also determined the rate of dissociation of insulin using advanced sampling techniques with two CVs: the separation between the center-of-mass of each monomer and the number of cross contacts between the C_*α*_ atoms of each monomer. The minimum free energy path for the dimer dissociation, the role of water, and in-depth analysis of transient conformers were investigated (131). These studies also elucidated the role of water in the structural stability of the insulin monomer, during dimer dissociation, and in maintaining the hexamer symmetry.

**Figure 7:**
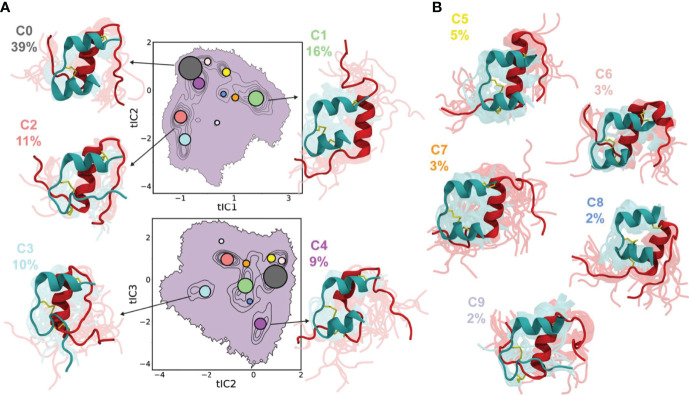
Conformational ensembles of the insulin monomer. Projections of ten conformers of the insulin monomer onto tIC1 vs tIC2 (top) and tIC2 vs tIC3 (bottom), with clusters indicated by contours. Each circle represents the median tIC values for a cluster, and the radius of circle is proportional to the physical weight of the cluster. Representative conformers for **(A)** first five clusters (C0–C4) constitute ~85% of the population and **(B)** the remaining clusters (C5–C9) constitute 15% of the population. The A-chain and B-chain of insulin are represented by teal and red cartoon representations, respectively. The abbreviation ‘tIC’ means time-lagged independent component. Reprinted from Busto-Moner et al. (132), an article published by American Chemical Society under the terms of the Creative Commons Attribution License (http://creativecommons.org/licenses/by/4.0/).

Not only MD simulations, a Markov state model (MSM) (172) was also constructed to probe the conformational dynamics of the insulin monomer (132) and the dimer (173) in aqueous solvent. Busto-Moner et al. (132) investigated the conformational heterogeneity of the human insulin monomer at pH 2.5 using MD simulations and enhanced sampling techniques in conjunction with integrated variational approach for conformational dynamics (IVAC), MSM, and Perron cluster analysis. They noticed 10 distinct conformational ensembles of the insulin monomer after conducting the clustering analysis (**Figure 7**). The most dominant cluster (accounting 38.7% of the total population) resembled the T state of insulin. The C-terminal helix in the A-chain and the helix formed by the LeuB15 to CysB19 residues were conserved in each cluster. The study also concluded that the A-chain N-terminal helix melting, detachment of the B-chain N-terminus, and the detachment of the B-chain C-terminus are atleast one of the structural disorders observed in 60% of total insulin monomer conformers. Feng et al. (173) also built an MSM from unbiased MD simulations to provide a microscopic view of conformational dynamics of the insulin dimer in aqueous water. The conformational transitions between the local and global conformations of the insulin dimer were further characterized with computational amide I spectroscopy (174). The study disclosed two significant conformations of the insulin dimer: a native state, and a twisted state (the B-chain helices of the native dimer interface rotated ~55° relative to each other). The MSM kinetics also reported that the transition of the native and twisted states of dimer occur on a 14 *µ*s time-scale. However, the significance of the twisted dimer state in dissociation of the insulin dimer remains elusive.

Santra et al. (129) performed MD simulations of insulin monomers in solutions of basic amino acids, namely, arginine, histidine, and lysine, to probe the stability of the protein. These solutions enhanced the stability of insulin over an aqueous solution. Among these three solutions, arginine provided the highest rigidity to the insulin structure followed by histidine and then lysine. A cluster analysis revealed that arginine, histidine, and lysine self-assembled to form larger clusters of sizes 16, 5, and 10 molecules, respectively. Arginine stabilized the insulin structure by forming relatively more hydrogen bonds and cation *π* interactions than histidine and lysine. The confined water molecules in the first solvation shell of insulin were least entropic in arginine solution than that of histidine and lysine solutions.

Sundaram et al. (128) elucidated the conformational stability of insulin aspart (ProB28 mutated to Asp residue) in aqueous cholinium aminoate ionic liquids comprised of different amino acids ([Ch][Gly], [Ch][Ala], and [Ch][Pro]) using MD simulations. Among these ionic liquids, the [Pro] anions of [Ch][Pro] established stable hydrogen bonds, coulombic and hydrophobic interactions, which provide structural integrity and conformational stability to insulin aspart. This study provided atomistic details of the interactions of [Ch][AA] with the insulin aspart and suggests the usage of ionic liquids for storing therapeutically-important proteins like insulin.

Using MD simulations in conjunction with the adiabatic-bias molecular dynamics (ABMD), Antoszewski et al. (133) reported six binding/unbinding pathways (PW1, PW2, PW3, PW4, PW1a, and PW4a) of phenol to/from the R_6_ insulin hexamer and its mutants (IleA10 to Val and GluB13 to Gln). This study demonstrated that the insulin hexamer is flexible and exhibits large-scale opening of a major escape channel to facilitate the dissociation of phenol molecules. The residues Ile10, His5, Leu13, and Leu17 were identified as gate-keeper residues near the phenol escape channels. The mutation of GluB13 to Gln residue was found to enhance phenol unbinding and stabilize the phenol-free state. Vashisth and Abrams (87) had earlier reported three pathways for phenol dissociation using multiple SMD simulations, but three new pathways were reported in this study (133): PW4, PW1a, and PW4a. The gate keeper residues corresponding to each pathway identified from both of these studies were consistent. The study by Antoszewski et al. (133) identified PW4a (phenol interacts with LeuA13 but not with LeuH17) pathway as the most preferred phenol dissociation pathway.

## 3 Summary

Starting with 1985 to date, we found 81 articles elucidating several crucial aspects of the insulin structure using MD simulation methods. In this section, we briefly list those research articles which were focused on the specific structural features of the hormone. The conformational dynamics of the insulin monomer, dimer, and hexamer in vacuum or in aqueous medium was investigated in several studies using energy minimizations and conventional MD simulations (56–58, 60–62, 69, 70, 73, 74, 77, 82, 86, 94, 97, 104, 110, 125). Enhanced sampling techniques were also extensively implemented to probe the equilibrium structural ensembles, folding/unfolding free-energy landscape, and to characterize the metastable ensembles along the transition pathway of the insulin monomer or dimer or hexamer (109, 117, 132, 173). Few studies specifically explored the transition between the T_6_↔R_6_ states of insulin using targeted (TEM and TMD) simulation methods (63, 64, 67, 68). The dissociation of the insulin dimer to its monomers is a crucial event in the hormone activity. The dissociation pathways, energetics, and thermodynamic parameters were characterized using simulation methods (66, 80, 81, 113, 115, 118, 119, 123, 124, 127, 130, 131).

Aggregation poses a major challenge in the storage and pharmacological activity of insulin. MD simulations were performed to understand the underlying mechanism of insulin aggregation and suggested few crucial residue mutations to impede the amyloid formation (90, 92, 96, 99). The B-chain of insulin seems to fold independently and plays an essential role during interaction with the IR. Thus, several studies were performed to understand the stability, flexibility, and conformational transitions of the B-chain of insulin using atomistic MD simulations (83, 85, 88). Specifically, the C-terminus of the B-chain of insulin behaves as a zipper and exhibits transitions between closed (inactive) to a wide-open (active) conformer to facilitate binding to IR. The conformational transitions, key residues, and the energetics of BC-CT were investigated using MD simulations (95, 100, 108). To probe the effect of varying pressure, temperature, pH, chemical modifications, and electric field on the structure of insulin, several MD studies were conducted (71, 78, 79, 84, 102, 122). The effect of cations, various green solvents, and small molecules on the conformational stability of insulin was explored using several simulation studies (52, 73, 76, 101, 103, 105, 111, 114, 120, 121, 126, 128, 129).

Phenol is often used as an antimicrobial agent to preserve the pharmaceutical insulin preparations and is known to stabilize the R_6 _state of the insulin hexamer. The dissociation pathways and their associated energy barriers were probed using extensive MD simulations (75, 87, 133). The stability and the activity of certain insulin analogs or chemically modified insulins using different MD simulation techniques were also reported (46, 59, 65, 93, 98, 125).

Outside of physiologically relevant simulation studies, we also found studies of insulin interactions with graphene, carbon nanotubes, material surfaces, and nanoparticles (89, 91, 106, 107, 112, 116). Apart from the articles summarized in this review, we observed a significant number of theoretical studies elucidating the mechanism of formation of amyloid fibrils, stability of insulin or its modified forms in various types of solvents or confined environments, and relevant to alternate insulin delivery, which we have classified as miscellaneous studies (76, 118, 175–202).

## 4 Outlook and Future Directions

Owing to several MD simulation studies described in this review, the key features of the structural stability, conformational transitions, dissociation/association energetics, and the activity of insulin are now fairly well understood. Moreover, the significance of the hydration shell and individual water molecules in the structural integrity of insulin monomer, dimer, and hexamer, and in the association/dissociation of insulin dimer have been probed in detail. It is becoming increasingly evident that the improved force-field parameters, better water models, enhanced sampling techniques, scalable simulation algorithms, and faster high-performance computing facilities have successfully expedited computational research in deciphering a wide array of structural features of insulin. Observing relevant conformational dynamics, collective motions, and protein-protein interactions may require longer timescales (203, 204). With the advancement of computational facilities and MD simulation techniques, longer timescale (ns to *μ*s) simulations of insulin were conducted to capture several biologically relevant events. For example, earlier simulations primarily investigated residue fluctuations and subtle conformational changes in the insulin monomer, dimer, and hexamer on a shorter timescale (56–58, 60, 62). However, in later studies, we observed simulations on longer simulation timescales and using enhanced sampling techniques to inspect the conformational heterogeneity, role of water molecules, and small molecules on the stability, association/dissociation energetics, and aggregation of insulin. The accuracy of MD simulations can be further enhanced using multiscale modeling, polarized water models, and polarized force fields (205–210). Moreover, the structure-prediction algorithms and platforms rooted in deep learning methods including AlphaFold (211) and RoseTTAFold (212) are gaining utility in solving protein structures and will prove useful to MD simulations as well.

MD simulations have complemented experimental observations and also played a crucial role in providing new molecular insights into the insulin structure and interactions. With the continuing advances in computing facilities, biomolecular force fields, and methodologies, we are likely to see significant progress in the application of MD simulations to understand the consequences of residue mutations on the stability, activity, and aggregation of insulin, and importantly in resolving the interactions of insulin with its cognate receptor. In this review, we have only discussed simulation studies conducted on the isolated insulin hormone or its self-assembled forms (dimer and hexamer), but to enrich our understanding of the interaction of insulin or its analogs with its cognate receptor, several MD simulation studies have been conducted (213–218), that were not discussed.

## Author Contributions

BG and HV contributed to conception and design of the review article. BG wrote the first draft of the manuscript. BG and HV contributed to manuscript revision and approved the submitted version.

## Funding

We acknowledge financial support from the National Institutes of Health (NIH) through grant R35GM138217 (HV). The content is solely the responsibility of the authors and does not necessarily represent the official views of the NIH.

## Conflict of Interest

The authors declare that the research was conducted in the absence of any commercial or financial relationships that could be construed as a potential conflict of interest.

## Publisher’s Note

All claims expressed in this article are solely those of the authors and do not necessarily represent those of their affiliated organizations, or those of the publisher, the editors and the reviewers. Any product that may be evaluated in this article, or claim that may be made by its manufacturer, is not guaranteed or endorsed by the publisher.

## Abbreviations

ABMD, Adiabatic-Bias Molecular Dynamics; aMD, accelerated molecular dynamics; BE-META, bias exchange metadynamics; CMEPS, computational mutations to estimate protein stability; D, insulin dimer; DPI, des-pentapeptide-(B26-B30)-insulin; DASI, diaminosuberoyl insulin (A1-B29 crosslinked); EM, energy minimization; H, insulin hexamer; IL, ionic liquid; IS, implicit solvent; M, insulin monomer; MM-GBSA, molecular mechanics-generalized Born surface area; MM-PBSA, molecular mechanics-Poisson Boltzmann surface area; MSM, Markov state model; n/a, not available; ProI, proinsulin; PTMetaD, parallel-tempering metadynamics; PTMetaD-WTE, parallel tempering metadynamics in a well-tempered ensemble; RAMD, random acceleration molecular dynamics; REMD, replica exchange molecular dynamics; REUS, Replica Exchange Umbrella Sampling; SA, simulated annealing; SMD, steered molecular dynamics; TEM, targeted energy minimization; TBS, tempered binding simulation; TMD, targeted molecular dynamics; TI, thermodynamic integration; US, umbrella sampling; PEG-M, Pegylated insulin monomer; WT-BEMD, well-tempered bias exchange metadynamics; WT-MD, well-tempered metadynamics.

## References

[1] Vecchio I Tornali C Bragazzi NL Martini M. The Discovery of Insulin: An Important Milestone in the History of Medicine. Front Endocrinol (2018) 9:613. doi: 10.3389/fendo.2018.00613 PMC6205949 30405529

[2] Hirsch IB Juneja R Beals JM Antalis CJ Wright EE Jr. The Evolution of Insulin and How it Informs Therapy and Treatment Choices. Endocrine Rev (2020) 41:733–55. doi: 10.1210/endrev/bnaa015 PMC7366348 32396624

[3] Flier JS Kahn CR. Insulin: A Pacesetter for the Shape of Modern Biomedical Science and the Nobel Prize. Mol Metab (2021) 52:101194. doi: 10.1016/j.molmet.2021.101194 33610859 PMC8513142

[4] Banting FG Best CH. The Internal Secretion of the Pancreas. J Lab Clin Med (1922) 7:465–80.

[5] Ward CW Lawrence MC. Landmarks in Insulin Research. Front Endocrinol (2011) 2:76. doi: 10.3389/fendo.2011.00076 PMC3356151 22654826

[6] Moroder L Musiol HJ. Insulin—from its Discovery to the Industrial Synthesis of Modern Insulin Analogues. Angewandte Chemie Int Edition (2017) 56:10656–69. doi: 10.1002/anie.201702493 28548452

[7] Alyas J Rafiq A Amir H Khan SU Sultana T Ali A. Human Insulin: History, Recent Advances, and Expression Systems for Mass Production. Biomed Res Ther (2021) 8:4540–61. doi: 10.15419/bmrat.v8i9.692

[8] Heller S Kozlovski P Kurtzhals P. Insulin’s 85th Anniversary—an Enduring Medical Miracle. Diabetes Res Clin Pract (2007) 78:149–58. doi: 10.1016/j.diabres.2007.04.001 17482306

[9] Stretton AO. The First Sequence: Fred Sanger and Insulin. Genetics (2002) 162:527–32. doi: 10.1093/genetics/162.2.527 PMC1462286 12399368

[10] Niu C Kung Y Huang W Ke L Chen C Chen Y. Synthesis of Peptide Fragments of the B-Chain of Insulin. Ix. Synthesis of the B-Chain of Insulin and Its Reconstitution with Natural A-Chain to Regenerate Insulin Activity. Scientia Sin (1964) 13:1343–5. 14206664

[11] Wang Y Hsu J Chang W Cheng L Li H Hsing C. Synthesis of A-Chain of Bovine Insulin and Partial Synthesis of Crystalline Bovine Insulin From Synthetic A-and Natural B-Chains. Acta Chim Sin (1966) 32:276–83.

[12] Qian YQ Tsou CL. Resynthesis of Insulin From its A and B Chains in the Presence of Denaturants. Biochem Biophys Res Commun (1987) 146:437–42. doi: 10.1016/0006-291X(87)90548-1 3304280

[13] Chance RE Frank BH. Research, Development, Production, and Safety of Biosynthetic Human Insulin. Diabetes Care (1993) 16:133–42. doi: 10.2337/diacare.16.3.133 8299470

[14] Adams MJ Blundell TL Dodson EJ Dodson GG Vijayan M Baker EN. Structure of Rhombohedral 2 Zinc Insulin Crystals. Nature (1969) 224:491–5. doi: 10.1038/224491a0

[15] Hirsch IB. Insulin Analogues. New Engl J Med (2005) 352:174–83. doi: 10.1056/NEJMra040832 15647580

[16] Mayer JP Zhang F DiMarchi RD. Insulin Structure and Function. Pept Science: Original Res Biomolecules (2007) 88:687–713. doi: 10.1002/bip.20734 17410596

[17] Weiss MA. The Structure and Function of Insulin: Decoding the Tr Transition. Vitamins Hormones (2009) 80:33–49. doi: 10.1016/S0083-6729(08)00602-X 19251033 PMC3297421

[18] Belgi A Akhter Hossain M W Tregear G D Wade J. The Chemical Synthesis of Insulin: From the Past to the Present. Immunology Endocrine Metab Agents Medicinal Chem (Formerly Curr Medicinal Chemistry-Immunology Endocrine Metab Agents) (2011) 11:40–7. doi: 10.2174/187152211794519412

[19] Mo R Jiang T Di J Tai W Gu Z. Emerging Micro-and Nanotechnology Based Synthetic Approaches for Insulin Delivery. Chem Soc Rev (2014) 43:3595–629. doi: 10.1039/c3cs60436e 24626293

[20] Tibaldi JM. Evolution of Insulin: From Human to Analog. Am J Med (2014) 127:S25–38. doi: 10.1016/j.amjmed.2014.07.005 25282010

[21] Vashisth H. Theoretical and Computational Studies of Peptides and Receptors of the Insulin Family. Membranes (2015) 5:48–83. doi: 10.3390/membranes5010048 25680077 PMC4384091

[22] Liu F Zaykov AN Levy JJ DiMarchi RD Mayer JP. Chemical Synthesis of Peptides Within the Insulin Superfamily. J Pept Sci (2016) 22:260–70. doi: 10.1002/psc.2863 26910514

[23] Belfiore A Malaguarnera R Vella V Lawrence MC Sciacca L Frasca F. Insulin Receptor Isoforms in Physiology and Disease: An Updated View. Endocrine Rev (2017) 38:379–431. doi: 10.1210/er.2017-00073 28973479 PMC5629070

[24] Selivanova O Grishin SY Glyakina A Sadgyan A Ushakova N Galzitskaya O. Analysis of Insulin Analogs and the Strategy of Their Further Development. Biochem (Moscow) (2018) 83:S146–62. doi: 10.1134/S0006297918140122 29544437

[25] Weiss MA Lawrence MC. A Thing of Beauty: Structure and Function of Insulin’s “Aromatic Triplet”. Diabet Obes Metab (2018) 20:51–63. doi: 10.1111/dom.13402 PMC6159917 30230175

[26] Sharma AK Taneja G Kumar A Sahu M Sharma G Kumar A. Insulin Analogs: Glimpse on Contemporary Facts and Future Prospective. Life Sci (2019) 219:90–9. doi: 10.1016/j.lfs.2019.01.011 30639280

[27] Rodbard HW Rodbard D. Biosynthetic Human Insulin and Insulin Analogs. Am J Ther (2020) 27:e42–51. doi: 10.1097/MJT.0000000000001089 31876563

[28] White MF Kahn CR. Insulin Action at a Molecular Level–100 Years of Progress. Mol Metab (2021) 52:101304. doi: 10.1016/j.molmet.2021.101304 34274528 PMC8551477

[29] Jarosinski MA Dhayalan B Chen YS Chatterjee D Varas N Weiss MA. Structural Principles of Insulin Formulation and Analog Design: A Century of Innovation. Mol Metab (2021) 52:101325. doi: 10.1016/j.molmet.2021.101325 34428558 PMC8513154

[30] Lizák B Szarka A Kim Y Ks C CE Németh Marcolongo P. Glucose Transport and Transporters in the Endomembranes. Int J Mol Sci (2019) 20:5898. doi: 10.3390/ijms20235898 PMC6929180 31771288

[31] Chadt A Al-Hasani H. Glucose Transporters in Adipose Tissue, Liver, and Skeletal Muscle in Metabolic Health and Disease. Pflügers Archiv-European J Physiol (2020) 472:1273–98. doi: 10.1007/s00424-020-02417-x PMC7462924 32591906

[32] Thorens B Mueckler M. Glucose Transporters in the 21st Century. Am J Physiology-Endocrinol Metab (2010) 298:E141–5. doi: 10.1152/ajpendo.00712.2009 PMC2822486 20009031

[33] Tan SY Wong JLM Sim YJ Wong SS Elhassan SAM Tan SH. Type 1 and 2 Diabetes Mellitus: A Review on Current Treatment Approach and Gene Therapy as Potential Intervention. Diabetes Metab Syndrome: Clin Res Rev (2019) 13:364–72. doi: 10.1016/j.dsx.2018.10.008 30641727

[34] Khawandanah J. Double or Hybrid Diabetes: A Systematic Review on Disease Prevalence, Characteristics and Risk Factors. Nutr Diabetes (2019) 9:1–9. doi: 10.1038/s41387-019-0101-1 31685799 PMC6828774

[35] Carroll RJ Hammer RE Chan SJ Swift HH Rubenstein AH Steiner DF. A Mutant Human Proinsulin is Secreted From Islets of Langerhans in Increased Amounts *via* an Unregulated Pathway. Proc Natl Acad Sci (1988) 85:8943–7. doi: 10.1073/pnas.85.23.8943 PMC282623 3057496

[36] Tanese T LAzARus NR Devrim S Recant L. Synthesis and Release of Proinsulin and Insulin by Isolated Rat Islets of Langerhans. J Clin Invest (1970) 49:1394–404. doi: 10.1172/JCI106357 PMC322613 4914679

[37] Weiss M Steiner DF Philipson LH. Insulin Biosynthesis, Secretion, Structure, and Structure-Activity Relationships. (2015). Available at: https://www.ncbi.nlm.nih.gov/books/NBK279029/. 25905258

[38] Das A Shah M Saraogi I. Molecular Aspects of Insulin Aggregation and Various Therapeutic Interventions. ACS Bio Med Chem Au (2022). doi: 10.1021/acsbiomedchemau.1c00054 PMC10114644 37101572

[39] Lawrence MC. Understanding Insulin and its Receptor From Their Three-Dimensional Structures. Mol Metab (2021) 52:101255. doi: 10.1016/j.molmet.2021.101255 33992784 PMC8513149

[40] De Meyts P. Insulin/receptor Binding: The Last Piece of the Puzzle? What Recent Progress on the Structure of the Insulin/Receptor Complex Tells Us (or Not) About Negative Cooperativity and Activation. Bioessays (2015) 37:389–97. doi: 10.1002/bies.201400190 25630923

[41] Brader M Kaarsholm N Dunn M. The R-State Proinsulin and Insulin Hexamers Mimic the Carbonic Anhydrase Active Site. J Biol Chem (1990) 265:15666–70. doi: 10.1016/S0021-9258(18)55450-8 2118529

[42] Rahuel-Clermont S French CA Kaarsholm NC Dunn MF. Mechanisms of Stabilization of the Insulin Hexamer Through Allosteric Ligand Interactions. Biochemistry (1997) 36:5837–45. doi: 10.1021/bi963038q 9153424

[43] Kosinová L Veverka V Novotná P Collinsová M Urbanová M Moody NR. Insight Into the Structural and Biological Relevance of the T/R Transition of the N-Terminus of the B-Chain in Human Insulin. Biochemistry (2014) 53:3392–402. doi: 10.1021/bi500073z PMC4047818 24819248

[44] Bentley G Dodson G Lewitova A. Rhombohedral Insulin Crystal Transformation. J Mol Biol (1978) 126:871–5. doi: 10.1016/0022-2836(78)90026-8 745246

[45] Adcock SA McCammon JA. Molecular Dynamics: Survey of Methods for Simulating the Activity of Proteins. Chem Rev (2006) 106:1589–615. doi: 10.1021/cr040426m PMC2547409 16683746

[46] Knegtel RM Boelens R Ganadu ML Kaptein R. The Solution Structure of a Monomeric Insulin: A Two-Dimensional 1H-NMR Study of des-(B26–B30)-Insulin in Combination With Distance Geometry and Restrained Molecular Dynamics. Eur J Biochem (1991) 202:447–58. doi: 10.1111/j.1432-1033.1991.tb16394.x 1761045

[47] Hua QX Gozani SN Chance RE Hoffmann JA Frank BH Weiss MA. Structure of a Protein in a Kinetic Trap. Nat Struct Biol (1995) 2:129–38. doi: 10.1038/nsb0295-129 7749917

[48] Bocian W Sitkowski J Bednarek E Tarnowska A Kawęcki R Kozerski L. Structure of Human Insulin Monomer in Water/Acetonitrile Solution. J Biomol NMR (2008) 40:55–64. doi: 10.1007/s10858-007-9206-2 18040865

[49] Whittingham JL Edwards DJ Antson AA Clarkson JM Dodson GG. Interactions of Phenol and m-Cresol in the Insulin Hexamer, and Their Effect on the Association Properties of B28 Pro→Asp Insulin Analogues. Biochemistry (1998) 37:11516–23. doi: 10.1021/bi980807s 9708987

[50] Yao ZP Zeng ZH Li HM Zhang Y Feng YM Wang DC. Structure of an Insulin Dimer in an Orthorhombic Crystal: The Structure Analysis of a Human Insulin Mutant (B9 Ser→ Glu). Acta Crystallograp (1999) 55:1524–32. doi: 10.1107/S0907444999008562 10489447

[51] Smith GD Pangborn WA Blessing RH. The Structure of T6 Bovine Insulin. Acta Crystallograp (2005) 61:1476–82. doi: 10.1107/S0907444905025771 16239724

[52] Palivec V Viola CM Kozak M Ganderton TR Křížková K Turkenburg JP. Computational and Structural Evidence for Neurotransmitter-Meiated Modulation of the Oligomeric States of Human Insulin in Storage Granules. J Biol Chem (2017) 292:8342–55. doi: 10.1074/jbc.M117.775924 PMC5437240 28348075

[53] Scapin G Dandey VP Zhang Z Prosise W Hruza A Kelly T. Structure of the Insulin Receptor–Insulin Complex by Single-Particle Cryo-Em Analysis. Nature (2018) 556(7699):122–5. doi: 10.1038/nature26153 PMC5886813 29512653

[54] Uchikawa E Choi E Shang G Yu H Bai Xc. Activation Mechanism of the Insulin Receptor Revealed by Cryo-Em Structure of the Fully Liganded Receptor–Ligand Complex. Elife (2019) 8:e48630. doi: 10.7554/eLife.48630.030 31436533 PMC6721835

[55] Gutmann T Schäfer IB Poojari C Brankatschk B Vattulainen I Strauss M. Cryo-Em Structure of the Complete and Ligand-Saturated Insulin Receptor Ectodomain. J Cell Biol (2020) 219:e201907210. doi: 10.1083/jcb.201907210 31727777 PMC7039211

[56] Wodak SJ Alard P Delhaise P Renneboog-Squilbin C. Simulation of Conformational Changes in 2 Zn Insulin. J Mol Biol (1985) 181:317–22. doi: 10.1016/0022-2836(85)90095-6 3884821

[57] Weiner SJ Kollman PA Nguyen DT Case DA. An All Atom Force Field for Simulations of Proteins and Nucleic Acids. J Comput Chem (1986) 7:230–52. doi: 10.1002/jcc.540070216 29160584

[58] Krüger P Strassburger W Wollmer A van Gunsteren W Dodson G. The Simulated Dynamics of the Insulin Monomer and Their Relationship to the Molecule’s Structure. Eur Biophys J (1987) 14:449–59. doi: 10.1007/BF00293254 3304990

[59] Yun-Yu S Ru-Huai Y Van Gunsteren W. Molecular Dynamics Simulation of Despentapeptide Insulin in a Crystalline Environment. J Mol Biol (1988) 200:571–7. doi: 10.1016/0022-2836(88)90543-8 3294422

[60] Caves LS Nguyen DT Hubbard RE. Conformational Variability of Insulin. Mol Dynam: Appl Mol Biol (1990) 16:27. doi: 10.1007/978-1-349-11044-5_2

[61] Badger J Caspar D. Water Structure in Cubic Insulin Crystals. Proc Natl Acad Sci (1991) 88:622–6. doi: 10.1073/pnas.88.2.622 PMC50864 1988957

[62] Mark AE Berendsen HJ Van Gunsteren WF. Conformational Flexibility of Aqueous Monomeric and Dimeric Insulin: A Molecular Dynamics Study. Biochemistry (1991) 30:10866–72. doi: 10.1021/bi00109a009 1932009

[63] Engels M Jacoby E Krüger P Schlitter J Wollmer A. The T↔R Structural Transition of Insulin; Pathways Suggested by Targeted Energy Minimization. Protein Engineer Des Select (1992) 5:669–77. doi: 10.1093/protein/5.7.669 1480621

[64] Schlitter J Engels M Krüger P Jacoby E Wollmer A. Targeted Molecular Dynamics Simulation of Conformational Change-Application to the T↔ R Transition in Insulin. Mol Simulat (1993) 10:291–308. doi: 10.1080/08927029308022170

[65] Krüger P Hahnen J Wollmer A. Comparative Studies on the Dynamics of Crosslinked Insulin. Eur Biophys J (1994) 23:177–87. doi: 10.1007/BF01007609 7956978

[66] Tidor B Karplus M. The Contribution of Vibrational Entropy to Molecular Association: The Dimerization of Insulin. J Mol Biol (1994) 238:405–14. doi: 10.1006/jmbi.1994.1300 8176732

[67] Schlitter J Engels M Krüger P. Targeted Molecular Dynamics: A New Approach for Searching Pathways of Conformational Transitions. J Mol Graphics (1994) 12:84–9. doi: 10.1016/0263-7855(94)80072-3 7918256

[68] Jacoby E Krüger P Schlitter J Röper D Wollmer A. Simulation of a Complex Protein Structural Change: The T↔ R Transition in the Insulin Hexamer. Protein Engineer Des Select (1996) 9:113–25. doi: 10.1093/protein/9.2.113 9005432

[69] Chang X Jørgensen AMM Bardrum P Led JJ. Solution Structures of the R_6_ Human Insulin Hexamer. Biochemistry (1997) 36:9409–22. doi: 10.1021/bi9631069 9235985

[70] Cheng YK Rossky PJ. The Effect of Vicinal Polar and Charged Groups on Hydrophobic Hydration. Biopol: Original Res Biomolecules (1999) 50:742–50. doi: 10.1002/(SICI)1097-0282(199912)50:7<742::AID-BIP7>3.0.CO;2-6 10547529

[71] Chai CC Jhon MS. Molecular Dynamics Study on Protein and It’s Water Structure at High Pressure. Mol Simulat (2000) 23:257–74. doi: 10.1080/08927020008025372

[72] Falconi M Bozzi M Paci M Raudino A Purrello R Cambria A. Spectroscopic and Molecular Dynamics Simulation Studies of the Interaction of Insulin With Glucose. Int J Biol Macromol (2001) 29:161–8. doi: 10.1016/S0141-8130(01)00157-X 11589968

[73] Falconi M Cambria MT Cambria A Desideri A. Structure and Stability of the Insulin Dimer Investigated by Molecular Dynamics Simulation. J Biomol Struct Dynam (2001) 18:761–72. doi: 10.1080/07391102.2001.10506705 11334112

[74] Wang WY Lu BZ Chen WZ Wang CX. Study on the Stability of Insulin Hexamer in Solution by Molecular Dynamics Simulations. Acta Chim Sinica-Chinese Edition- (2002) 60:2129–34.

[75] Swegat W Schlitter J Krüger P Wollmer A. MD Simulation of Protein-Ligand Interaction: Formation and Dissociation of an Insulin-Phenol Complex. Biophys J (2003) 84:1493–506. doi: 10.1016/S0006-3495(03)74962-5 PMC1302723 12609856

[76] Zoete V Meuwly M Karplus M. Investigation of Glucose Binding Sites on Insulin. Proteins (2004) 55:568–81. doi: 10.1002/prot.20071 15103621

[77] Zoete V Meuwly M Karplus M. A Comparison of the Dynamic Behavior of Monomeric and Dimeric Insulin Shows Structural Rearrangements in the Active Monomer. J Mol Biol (2004) 342:913–29. doi: 10.1016/j.jmb.2004.07.033 15342246

[78] Budi A Legge S Treutlein H Yarovsky I. Effect of External Stresses on Protein Conformation: A Computer Modelling Study. Eur Biophys J (2004) 33:121–9. doi: 10.1007/s00249-003-0359-y 14574523

[79] Budi A Legge FS Treutlein H Yarovsky I. Electric Field Effects on Insulin Chain-B Conformation. J Phys Chem B (2005) 109:22641–8. doi: 10.1021/jp052742q 16853947

[80] Zoete V Meuwly M Karplus M. Study of the Insulin Dimerization: Binding Free Energy Calculations and Per-Residue Free Energy Decomposition. Proteins (2005) 61:79–93. doi: 10.1002/prot.20528 16080143

[81] Kim T Rhee A Yip CM. Force-Induced Insulin Dimer Dissociation: A Molecular Dynamics Study. J Am Chem Soc (2006) 128:5330–1. doi: 10.1021/ja0607382 16620090

[82] Zoete V Meuwly M. Importance of Individual Side Chains for the Stability of a Protein Fold: Computational Alanine Scanning of the Insulin Monomer. J Comput Chem (2006) 27:1843–57. doi: 10.1002/jcc.20512 16981237

[83] Legge F Budi A Treutlein H Yarovsky I. Protein Flexibility: Multiple Molecular Dynamics Simulations of Insulin Chain B. Biophys Chem (2006) 119:146–57. doi: 10.1016/j.bpc.2005.08.002 16129550

[84] Budi A Legge FS Treutlein H Yarovsky I. Effect of Frequency on Insulin Response to Electric Field Stress. J Phys Chem B (2007) 111:5748–56. doi: 10.1021/jp067248g 17472363

[85] Budi A Legge FS Treutlein H Yarovsky I. Comparative Study of Insulin Chain-B in Isolated and Monomeric Environments Under External Stress. J Phys Chem B (2008) 112:7916–24. doi: 10.1021/jp800350v 18537286

[86] Todorova N Legge FS Treutlein H Yarovsky I. Systematic Comparison of Empirical Forcefields for Molecular Dynamic Simulation of Insulin. J Phys Chem B (2008) 112:11137–46. doi: 10.1021/jp076825d 18698702

[87] Vashisth H Abrams CF. Ligand Escape Pathways and (Un) Binding Free Energy Calculations for the Hexameric Insulin-Phenol Complex. Biophys J (2008) 95:4193–204. doi: 10.1529/biophysj.108.139675 PMC2567929 18676643

[88] Todorova N Marinelli F Piana S Yarovsky I. Exploring the Folding Free Energy Landscape of Insulin Using Bias Exchange Metadynamics. J Phys Chem B (2009) 113:3556–64. doi: 10.1021/jp809776v 19243106

[89] Shen JW Wu T Wang Q Kang Y Chen X. Adsorption of Insulin Peptide on Charged Single-Walled Carbon Nanotubes: Significant Role of Ordered Water Molecules. ChemPhysChem (2009) 10:1260–9. doi: 10.1002/cphc.200800836 19353602

[90] Haas J Vöhringer-Martinez E Bögehold A Matthes D Hensen U Pelah A. Primary Steps of Ph-Dependent Insulin Aggregation Kinetics are Governed by Conformational Flexibility. ChemBioChem (2009) 10:1816–22. doi: 10.1002/cbic.200900266 19533727

[91] Liang Lj Wang Q Wu T Jw S Kang Y. Molecular Dynamics Simulation on Stability of Insulin on Graphene. Chin J Chem Phys (2009) 22:627. doi: 10.1088/1674-0068/22/06/627-634

[92] Todorova N Yarovsky I. Molecular Modelling of Peptide Folding, Misfolding and Aggregation Phenomena. Proc Comput Sci (2010) 1:1185–93. doi: 10.1016/j.procs.2010.04.132

[93] Yang C Lu D Liu Z. How Pegylation Enhances the Stability and Potency of Insulin: A Molecular Dynamics Simulation. Biochemistry (2011) 50:2585–93. doi: 10.1021/bi101926u 21332191

[94] Yamamoto S Kaminský J Bouř P. Structure and Vibrational Motion of Insulin From Raman Optical Activity Spectra. Anal Chem (2012) 84:2440–51. doi: 10.1021/ac2032436 22263577

[95] Žáková L Kletvíková E Veverka V Lepšík M Watson CJ Turkenburg JP. Structural Integrity of the B24 Site in Human Insulin is Important for Hormone Functionality. J Biol Chem (2013) 288:10230–40. doi: 10.1074/jbc.M112.448050 PMC3624407 23447530

[96] Chinisaz M Larijani B Ebrahim-Habibi A. A Molecular Modeling Study on Full-Length Insulin: Insight Into Initial Events of Amyloid Formation. Struct Chem (2014) 25:1175–85. doi: 10.1007/s11224-014-0395-5

[97] Bagchi K Roy S. Sensitivity of Water Dynamics to Biologically Significant Surfaces of Monomeric Insulin: Role of Topology and Electrostatic Interactions. J Phys Chem B (2014) 118:3805–13. doi: 10.1021/jp411136w 24641444

[98] Kim YH Kastner K Abdul-Wahid B Izaguirre JA. Evaluation of Conformational Changes in Diabetes-Associated Mutation in Insulin a Chain: A Molecular Dynamics Study. Proteins (2015) 83:662–9. doi: 10.1002/prot.24759 PMC4382306 25641314

[99] Mishra NK Krishna Deepak R Sankararamakrishnan R Verma S. Controlling *In Vitro* Insulin Amyloidosis With Stable Peptide Conjugates: A Combined Experimental and Computational Study. J Phys Chem B (2015) 119:15395–406. doi: 10.1021/acs.jpcb.5b08215 26569375

[100] Papaioannou A Kuyucak S Kuncic Z. Molecular Dynamics Simulations of Insulin: Elucidating the Conformational Changes That Enable its Binding. PLoS One (2015) 10:e0144058. doi: 10.1371/journal.pone.0144058 26629689 PMC4668001

[101] Muhammad EF Adnan R Latif MAM Abdul Rahman MB. Theoretical Investigation on Insulin Dimer *β*-Cyclodextrin Interactions Using Docking and Molecular Dynamics Simulation. J Inclusion Phenomena Macrocyclic Chem (2016) 84:1–10. doi: 10.1007/s10847-015-0576-x

[102] Baheri M Dayer MR. Temperature and pH Effects on Insulin Structure: A Molecular Dynamic Approach. Jentashapir J Health Res (2016) 7:e36931. doi: 10.17795/jjhr-36931

[103] Soleymani H Saboury AA Moosavi-Movahedi AA Rahmani F Maleki J Yousefinejad S. Vitamin E Induces Regular Structure and Stability of Human Insulin, More Intense Than Vitamin D_3_ . Int J Biol Macromol (2016) 93:868–78. doi: 10.1016/j.ijbiomac.2016.09.047 27642128

[104] Sklepari M Rodger A Reason A Jamshidi S Prokes I Blindauer CA. Biophysical Characterization of a Protein for Structure Comparison: Methods for Identifying Insulin Structural Changes. Anal Methods (2016) 8:7460–71. doi: 10.1039/C6AY01573E

[105] Pan X Li D Wei D. Structural Stability of Insulin in Imidazolium Ionic Liquids by Molecular Simulation. CIESC J (2016) 67:5215–21. doi: 10.11949/j.issn.0438-1157.20160968

[106] Nejad MA Mücksch C Urbassek HM. Insulin Adsorption on Crystalline Sio2: Comparison Between Polar and Nonpolar Surfaces Using Accelerated Molecular-Dynamics Simulations. Chem Phys Lett (2017) 670:77–83. doi: 10.1016/j.cplett.2017.01.002

[107] Atabay M Jahanbin Sardroodi J Rastkar Ebrahimzadeh A. Adsorption and Immobilisation of Human Insulin on Graphene Monoxide, Silicon Carbide and Boron Nitride Nanosheets Investigated by Molecular Dynamics Simulation. Mol Simulat (2017) 43:298–311. doi: 10.1080/08927022.2016.1270452

[108] Papaioannou A Kuyucak S Kuncic Z. Computational Study of the Activity, Dynamics, Energetics and Conformations of Insulin Analogues Using Molecular Dynamics Simulations: Application to Hyperinsulinemia and the Critical Residue B26. Biochem Biophys Rep (2017) 11:182–90. doi: 10.1016/j.bbrep.2017.04.006 PMC5614686 28955783

[109] Singh R Bansal R Rathore AS Goel G. Equilibrium Ensembles for Insulin Folding From Bias-Exchange Metadynamics. Biophys J (2017) 112:1571–85. doi: 10.1016/j.bpj.2017.03.015 PMC5406382 28445749

[110] Mukherjee S Mondal S Deshmukh AA Gopal B Bagchi B. What Gives an Insulin Hexamer its Unique Shape and Stability? Role of Ten Confined Water Molecules. J Phys Chem B (2018) 122:1631–7. doi: 10.1021/acs.jpcb.8b00453 29341613

[111] Duboué-Dijon E Delcroix P Martinez-Seara H Hladílková J Coufal P Křížek T. Binding of Divalent Cations to Insulin: Capillary Electrophoresis and Molecular Simulations. J Phys Chem B (2018) 122:5640–8. doi: 10.1021/acs.jpcb.7b12097 29360367

[112] Nejad MA Urbassek HM. Insulin Adsorption on Functionalized Silica Surfaces: An Accelerated Molecular Dynamics Study. J Mol Model (2018) 24:1–7. doi: 10.1007/s00894-018-3610-2 29524008

[113] Raghunathan S El Hage K Desmond JL Zhang L Meuwly M. The Role of Water in the Stability of Wild-Type and Mutant Insulin Dimers. J Phys Chem B (2018) 122:7038–48. doi: 10.1021/acs.jpcb.8b04448 29916244

[114] Březina K Duboué-Dijon E Palivec V Jiráček J Křížek T Viola CM. Can Arginine Inhibit Insulin Aggregation? A Combined Protein Crystallography, Capillary Electrophoresis, and Molecular Simulation Study. J Phys Chem B (2018) 122:10069–76. doi: 10.1021/acs.jpcb.8b06557 30153414

[115] Banerjee P Mondal S Bagchi B. Insulin Dimer Dissociation in Aqueous Solution: A Computational Study of Free Energy Landscape and Evolving Microscopic Structure Along the Reaction Pathway. J Chem Phys (2018) 149:114902. doi: 10.1063/1.5042290 30243274

[116] Hosseinzadeh G Maghari A Farnia SMF Moosavi-Movahedi AA. Interaction Mechanism of Insulin With ZnO Nanoparticles by Replica Exchange Molecular Dynamics Simulation. J Biomol Struct Dynam (2018) 36:3623–35. doi: 10.1080/07391102.2017.1396254 29064322

[117] Mukherjee S Deshmukh AA Mondal S Gopal B Bagchi B. Destabilization of Insulin Hexamer in Water–Ethanol Binary Mixture. J Phys Chem B (2019) 123:10365–75. doi: 10.1021/acs.jpcb.9b07689 31726824

[118] Pan AC Jacobson D Yatsenko K Sritharan D Weinreich TM Shaw DE. Atomic-Level Characterization of Protein–Protein Association. Proc Natl Acad Sci (2019) 116:4244–9. doi: 10.1073/pnas.1815431116 PMC6410769 30760596

[119] Banerjee P Mondal S Bagchi B. Effect of Ethanol on Insulin Dimer Dissociation. J Chem Phys (2019) 150:084902. doi: 10.1063/1.5079501 30823756

[120] Li D Gao Y Pan X Wei D Guo B Yang C. Md and Dsc Study of Bioactive Structural Stability of Insulin in Various Imidazolium Ionic Liquids. J Mol Liquids (2019) 277:971–6. doi: 10.1016/j.molliq.2019.01.039

[121] Soleymani H Ghorbani M Allahverdi A Shojaeilangari S Naderi-Manesh H. Activation of Human Insulin by Vitamin E: A Molecular Dynamics Simulation Study. J Mol Graphics Model (2019) 91:194–203. doi: 10.1016/j.jmgm.2019.06.006 31265936

[122] Kurpiewska K Miłaczewska A Lewiński K. Insulin Conformational Changes Under High Pressure in Structural Studies and Molecular Dynamics Simulations. J Mol Struct (2020) 1202:127251. doi: 10.1016/j.molstruc.2019.127251

[123] Gong Q Zhang H Zhang H Chen C. Calculating the Absolute Binding Free Energy of the Insulin Dimer in an Explicit Solvent. RSC Adv (2020) 10:790–800. doi: 10.1039/C9RA08284K 35494470 PMC9047981

[124] Banerjee P Bagchi B. Dynamical Control by Water at a Molecular Level in Protein Dimer Association and Dissociation. Proc Natl Acad Sci (2020) 117:2302–8. doi: 10.1073/pnas.1908379117 PMC7007538 31969453

[125] Yusuff OK Raji AT Abdul Raheem MAO Ojo DB. Explicit Solvent Molecular Dynamics Simulation Studies of the Dissociation of Human Insulin Hexamer Into the Dimeric Units. Adv J Chemistry-Section A (2020) 3:730–9. doi: 10.22034/AJCA.2020.107265

[126] Santra S Jana M. Insights Into the Sensitivity of Arginine Concentration to Preserve the Folded Form of Insulin Monomer Under Thermal Stress. J Chem Inf Model (2020) 60:3105–19. doi: 10.1021/acs.jcim.0c00006 32479724

[127] Antoszewski A Feng CJ Vani BP Thiede EH Hong L Weare J. Insulin Dissociates by Diverse Mechanisms of Coupled Unfolding and Unbinding. J Phys Chem B (2020) 124:5571–87. doi: 10.1021/acs.jpcb.0c03521 PMC7774804 32515958

[128] Sundaram V Ramanan RN Selvaraj M Vijayaraghavan R MacFarlane DR Ooi CW. Structural Stability of Insulin Aspart in Aqueous Cholinium Aminoate Ionic Liquids Based on Molecular Dynamics Simulation Studies. J Mol Liquids (2021) 322:114501. doi: 10.1016/j.molliq.2020.114501

[129] Santra S Dhurua S Jana M. Analyzing the Driving Forces of Insulin Stability in the Basic Amino Acid Solutions: A Perspective From Hydration Dynamics. J Chem Phys (2021) 154:084901. doi: 10.1063/5.0038305 33639734

[130] Mondal S Mukherjee S Acharya S Bagchi B. Unfolding of Dynamical Events in the Early Stage of Insulin Dimer Dissociation. J Phys Chem B (2021) 125:7958–66. doi: 10.1021/acs.jpcb.1c03104 34260242

[131] Acharya S Mondal S Mukherjee S Bagchi B. Rate of Insulin Dimer Dissociation: Interplay Between Memory Effects and Higher Dimensionality. J Phys Chem B (2021) 125:9678–91. doi: 10.1021/acs.jpcb.1c03779 34406771

[132] Busto-Moner L Feng CJ Antoszewski A Tokmakoff A Dinner AR. Structural Ensemble of the Insulin Monomer. Biochemistry (2021) 60:3125–36. doi: 10.1021/acs.biochem.1c00583 PMC8552439 34637307

[133] Antoszewski A Lorpaiboon C Strahan J Dinner AR. Kinetics of Phenol Escape From the Insulin R_6_ Hexamer. J Phys Chem B (2021) 125:11637–49. doi: 10.1021/acs.jpcb.1c06544 PMC8878557 34648712

[134] Van Gunsteren WF Berendsen HJ. Computer Simulation of Molecular Dynamics: Methodology, Applications, and Perspectives in Chemistry. Angewandte Chemie Int Edition English (1990) 29:992–1023. doi: 10.1002/anie.199009921

[135] MacKerell AD Jr. Bashford D Bellott M Dunbrack RL Jr. Evanseck JD Field MJ. All-Atom Empirical Potential for Molecular Modeling and Dynamics Studies of Proteins. J Phys Chem B (1998) 102:3586–616. doi: 10.1021/jp973084f 24889800

[136] Karplus M McCammon JA. Molecular Dynamics Simulations of Biomolecules. Nat Struct Biol (2002) 9:646–52. doi: 10.1038/nsb0902-646 12198485

[137] Durrant JD McCammon JA. Molecular Dynamics Simulations and Drug Discovery. BMC Biol (2011) 9:1–9. doi: 10.1186/1741-7007-9-71 22035460 PMC3203851

[138] Schlick T Collepardo-Guevara R Halvorsen LA Jung S Xiao X. Biomolecular Modeling and Simulation: A Field Coming of Age. Q Rev Biophys (2011) 44:191–228. doi: 10.1017/S0033583510000284 21226976 PMC3700731

[139] Dror RO Dirks RM Grossman J Xu H Shaw DE. Biomolecular Simulation: A Computational Microscope for Molecular Biology. Annu Rev Biophys (2012) 41:429–52. doi: 10.1146/annurev-biophys-042910-155245 22577825

[140] Laage D Elsaesser T Hynes JT. Water Dynamics in the Hydration Shells of Biomolecules. Chem Rev (2017) 117:10694–725. doi: 10.1021/acs.chemrev.6b00765 PMC5571470 28248491

[141] Hollingsworth SA Dror RO. Molecular Dynamics Simulation for All. Neuron (2018) 99:1129–43. doi: 10.1016/j.neuron.2018.08.011 PMC6209097 30236283

[142] Huggins DJ Biggin PC Dämgen MA Essex JW Harris SA Henchman RH. Biomolecular Simulations: From Dynamics and Mechanisms to Computational Assays of Biological Activity. Wiley Interdiscip Rev: Comput Mol Sci (2019) 9:e1393. doi: 10.1002/wcms.1393

[143] Schlick T Portillo-Ledesma S Myers CG Beljak L Chen J Dakhel S. Biomolecular Modeling and Simulation: A Prospering Multidisciplinary Field. Annu Rev Biophys (2021) 50:267–301. doi: 10.1146/annurev-biophys-091720-102019 33606945 PMC8105287

[144] Berendsen HJ Postma JV Van Gunsteren WF DiNola AR Haak JR. Molecular Dynamics With Coupling to an External Bath. J Chem Phys (1984) 81:3684–90. doi: 10.1063/1.448118

[145] Van Gunsteren W Berendsen HJ. Algorithms for Macromolecular Dynamics and Constraint Dynamics. Mol Phys (1977) 34:1311–27. doi: 10.1080/00268977700102571

[146] Berendsen HJC Postma JPM van Gunsteren WF Hermans J. Interaction Models for Water in Relation to Protein Hydration. *Intermol Force (Springer)* Pullman B. Intermolecular Forces: Proceedings of the Fourteenth Jerusalem Symposium on Quantum Chemistry and Biochemistry Held in Jerusalem, Israel, April 13--16, 1981. Dordrecht: Springer (1981), 331–42. doi: 10.1007/978-94-015-7658-1_21

[147] Baker EN Blundell TL Cutfield JF Dodson EJ Dodson GG Hodgkin DMC. The Structure of 2zn Pig Insulin Crystals at 1.5 Å Resolution. Philosophical Transactions of the Royal Society of London. B Biol Sci (1988) 319:369–456. doi: 10.1098/rstb.1988.0058 2905485

[148] Caspar D Clarage J Salunke D Clarage M. Liquid-Like Movements in Crystalline Insulin. Nature (1988) 332:659–62. doi: 10.1038/332659a0 3282173

[149] Dodson E Dodson G Hubbard R Reynolds C. Insulin’s Structural Behavior and its Relation to Activity. Biopol: Original Res Biomolecules (1983) 22:281–91. doi: 10.1002/bip.360220137 6370324

[150] Weiss MA Nguyen DT Khait I Inouye K Frank BH Beckage M. Two-Dimensional NMR and Photo-CIDNP Studies of the Insulin Monomer: Assignment of Aromatic Resonances With Application to Protein Folding, Structure, and Dynamics. Biochemistry (1989) 28:9855–73. doi: 10.1021/bi00451a046 2692717

[151] Strazza S Hunter R Walker E Darnall DW. The Thermodynamics of Bovine and Porcine Insulin and Proinsulin Association Determined by Concentration Difference Spectroscopy. Arch Biochem Biophys (1985) 238:30–42. doi: 10.1016/0003-9861(85)90137-7 3885857

[152] Brandenburg D Busse WD Gattner HG Zahn H Wollmer A Gliemann J. Structure-Function Studies With Chemically Modified Insulins. In: Proceedings of the Twelfth European Peptide Symposium. Amsterdam: North Holland Publishing Co. (1973). pp. 270–284.

[153] Lindsay D. Intramolecular Cross-Linked Insulin. FEBS Lett (1972) 21:105–8. doi: 10.1016/0014-5793(72)80175-3 11946487

[154] Nakagawa SH Tager H. Perturbation of Insulin-Receptor Interactions by Intramolecular Hormone Cross-Linking: Analysis of Relative Movement Among Residues A1, B1, and B29. J Biol Chem (1989) 264:272–9. doi: 10.1016/S0021-9258(17)31254-1 2642474

[155] Brems D Brown P Nakagawa S Tager H. The Conformational Stability and Flexibility of Insulin With an Additional Intramolecular Cross-Link. J Biol Chem (1991) 266:1611–5. doi: 10.1016/S0021-9258(18)52338-3 1988440

[156] Markussen J Jørgensen KH Sørensen AR Thim L. Single Chain Des-(B30) Insulin: Intramolecular Crosslinking of Insulin by Trypsin Catalyzed Transpeptidation. Int J Pept Protein Res (1985) 26:70–7. doi: 10.1111/j.1399-3011.1985.tb03179.x 3902689

[157] Derewenda U Derewenda Z Dodson EJ Dodson GG Bing X Markussen J. X-Ray Analysis of the Single Chain B29-A1 Peptide-Linked Insulin Molecule: A Completely Inactive Analogue. J Mol Biol (1991) 220(2):425–33. doi: 10.1016/0022-2836(91)90022-x 1856866

[158] Brange J Owens DR Kang S Vølund A. Monomeric Insulins and Their Experimental and Clinical Implications. Diabetes Care (1990) 13:923–54. doi: 10.2337/diacare.13.9.923 2226110

[159] Brange J Dodson GG Xiao B. Designing Insulin for Diabetes Therapy by Protein Engineering. Curr Opin Struct Biol (1991) 1:934–40. doi: 10.1016/0959-440X(91)90088-B

[160] Hawkins B Cross K Craik D. Solution Structure of the B-Chain of Insulin as Determined by 1H NMR Spectroscopy Comparison With the Crystal Structure of the Insulin Hexamer and With the Solution Structure of the Insulin Monomer. Int J Pept Protein Res (1995) 46:424–33. doi: 10.1111/j.1399-3011.1995.tb01077.x 8567187

[161] Hua QX Nakagawa SH Jia W Hu SQ Chu YC Katsoyannis PG. Hierarchical Protein Folding: Asymmetric Unfolding of an Insulin Analogue Lacking the A7-B7 Interchain Disulfide Bridge. Biochemistry (2001) 40:12299–311. doi: 10.1021/bi011021o 11591149

[162] Laio A Parrinello M. Escaping Free-Energy Minima. Proc Natl Acad Sci (2002) 99:12562–6. doi: 10.1073/pnas.202427399 PMC130499 12271136

[163] Barducci A Bonomi M Parrinello M. Metadynamics. Wiley Interdiscip Rev: Comput Mol Sci (2011) 1:826–43. doi: 10.1002/wcms.31

[164] Piana S Laio A. A Bias-Exchange Approach to Protein Folding. J Phys Chem B (2007) 111:4553–9. doi: 10.1021/jp067873l 17419610

[165] Ward CW Menting JG Lawrence MC. The Insulin Receptor Changes Conformation in Unforeseen Ways on Ligand Binding: Sharpening the Picture of Insulin Receptor Activation. Bioessays (2013) 35:945–54. doi: 10.1002/bies.201300065 24037759

[166] Žáková L Kletvíková E Lepšík M Collinsová M Watson CJ Turkenburg JP. Human Insulin Analogues Modified at the B26 Site Reveal a Hormone Conformation That is Undetected in the Receptor Complex. Acta Crystallograp Sect D: Biol Crystallogr (2014) 70:2765–74. doi: 10.1107/S1399004714017775 PMC4188015 25286859

[167] Menting JG Yang Y Chan SJ Phillips NB Smith BJ Whittaker J. Protective Hinge in Insulin Opens to Enable its Receptor Engagement. Proc Natl Acad Sci (2014) 111:E3395–404. doi: 10.1073/pnas.1412897111 PMC4143003 25092300

[168] Lindorff-Larsen K Piana S Palmo K Maragakis P Klepeis JL Dror RO. Improved Side-Chain Torsion Potentials for the Amber ff99sb Protein Force Field. Proteins (2010) 78:1950–8. doi: 10.1002/prot.22711 PMC2970904 20408171

[169] Martinek T Duboué-Dijon E Timr Š Mason PE Baxová K Fischer HE. Calcium Ions in Aqueous Solutions: Accurate Force Field Description Aided by Ab Initio Molecular Dynamics and Neutron Scattering. J Chem Phys (2018) 148:222813. doi: 10.1063/1.5006779 29907056

[170] Hosseinzadeh G Maghari A Saboury AA Moosavi-Movahedi AA. Unfolding of Insulin at the Surface of ZnO Quantum Dots. Int J Biol Macromol (2016) 86:169–76. doi: 10.1016/j.ijbiomac.2016.01.075 26812116

[171] Mukherjee S Acharya S Mondal S Banerjee P Bagchi B. Structural Stability of Insulin Oligomers and Protein Association–Dissociation Processes: Free Energy Landscape and Universal Role of Water. J Phys Chem B (2021) 125:11793–811. doi: 10.1021/acs.jpcb.1c05811 34674526

[172] Husic BE Pande VS. Markov State Models: From an Art to a Science. J Am Chem Soc (2018) 140:2386–96. doi: 10.1021/jacs.7b12191 29323881

[173] Feng CJ Sinitskiy A Pande V Tokmakoff A. Computational IR Spectroscopy of Insulin Dimer Structure and Conformational Heterogeneity. J Phys Chem B (2021) 125:4620–33. doi: 10.1021/acs.jpcb.1c00399 PMC8442834 33929849

[174] Reppert M Tokmakoff A. Computational Amide I 2D IR Spectroscopy as a Probe of Protein Structure and Dynamics. Annu Rev Phys Chem (2016) 67:359–86. doi: 10.1146/annurev-physchem-040215-112055 27023758

[175] El-Refai M Bergman RN. Simulation Study of Control of Hepatic Glycogen Synthesis by Glucose and Insulin. Am J Physiology-Legacy Content (1976) 231:1608–19. doi: 10.1152/ajplegacy.1976.231.5.1608 187069

[176] Greaves RB Dodson GG Verma CS. Altered Ionization of the B13 Glu in Insulin B9 and B10 Mutants: A Computational Analysis. Protein Eng Des Select (2004) 17:557–63. doi: 10.1093/protein/gzh066 15326283

[177] Petrus AK Allis DG Smith RP Fairchild TJ Doyle RP. Exploring the Implications of Vitamin B12 Conjugation to Insulin on Insulin Receptor Binding. ChemMedChem: Chem Enabling Drug Discovery (2009) 4:421–6. doi: 10.1002/cmdc.200800346 19101970

[178] Pinero-González J González-Pérez A. The Ubiquity of the Insulin Superfamily Across the Eukaryotes Detected Using a Bioinformatics Approach. OMICS: A J Integr Biol (2011) 15:439–47. doi: 10.1089/omi.2010.0141 21410328

[179] Schor M Vreede J Bolhuis PG. Elucidating the Locking Mechanism of Peptides Onto Growing Amyloid Fibrils Through Transition Path Sampling. Biophys J (2012) 103:1296–304. doi: 10.1016/j.bpj.2012.07.056 PMC3446680 22995502

[180] Kitagawa Y Akinaga Y Kawashima Y Jung J Ten-no S. A QM/MM-MD Study on Protein Electronic Properties: Circular Dichroism Spectra of Oxytocin and Insulin. Chem Phys (2012) 401:95–102. doi: 10.1016/j.chemphys.2011.10.022

[181] Berhanu WM Masunov AE. Controlling the Aggregation and Rate of Release in Order to Improve Insulin Formulation: Molecular Dynamics Study of Full-Length Insulin Amyloid Oligomer Models. J Mol Model (2012) 18:1129–42. doi: 10.1007/s00894-011-1123-3 21674205

[182] Kurouski D Washington J Ozbil M Prabhakar R Shekhtman A Lednev IK. Disulfide Bridges Remain Intact While Native Insulin Converts Into Amyloid Fibrils. PLoS One (2012) 7:e36989. doi: 10.1371/journal.pone.0036989 22675475 PMC3365881

[183] Hong Y Meng L Chen S Leung CWT Da LT Faisal M. Monitoring and Inhibition of Insulin Fibrillation by a Small Organic Fluorogen With Aggregation-Induced Emission Characteristics. J Am Chem Soc (2012) 134:1680–9. doi: 10.1021/ja208720a 22191699

[184] da Cruz CH Seabra G. Molecular Dynamics Simulations Reveal a Novel Mechanism for ATP Inhibition of Insulin Degrading Enzyme. J Chem Inf Model (2014) 54:1380–90. doi: 10.1021/ci400695m 24697863

[185] Chiang HL Ngo ST Chen CJ Hu CK Li MS. Oligomerization of Peptides LVEALYL and RGFFYT and Their Binding Affinity to Insulin. PLoS One (2013) 8:e65358. doi: 10.1371/journal.pone.0065358 23805182 PMC3689759

[186] Islam MA Bhayye S Adeniyi AA Soliman ME Pillay TS. Diabetes Mellitus Caused by Mutations in Human Insulin: Analysis of Impaired Receptor Binding of Insulins Wakayama, Los Angeles and Chicago Using Pharmacoinformatics. J Biomol Struct Dynam (2017) 35:724–37. doi: 10.1080/07391102.2016.1160258 26950281

[187] Amdursky N Rashid MH Stevens MM Yarovsky I. Exploring the Binding Sites and Proton Diffusion on Insulin Amyloid Fibril Surfaces by Naphthol-Based Photoacid Fluorescence and Molecular Simulations. Sci Rep (2017) 7:1–12. doi: 10.1038/s41598-017-06030-4 28740173 PMC5524688

[188] Chinisaz M Ebrahim-Habibi A Dehpour AR Yaghmaei P Parivar K Moosavi-Movahedi AA. Structure and Function of Anhydride-Modified Forms of Human Insulin: *In Silico*, *In Vitro* and *In Vivo* Studies. Eur J Pharm Sci (2017) 96:342–50. doi: 10.1016/j.ejps.2016.09.030 27687638

[189] Lei Y He J Liu J Li J. Conformational Ensemble of B Chain in T_6_ Human Insulin Based on the Landau Free Energy. Acta Physica Polonica A (2018) 133:1261–65. doi: 10.12693/APhysPolA.133.1261

[190] Rege NK Wickramasinghe NP Tustan AN Phillips NF Yee VC Ismail-Beigi F. Structure-Based Stabilization of Insulin as a Therapeutic Protein Assembly *via* Enhanced Aromatic–Aromatic Interactions. J Biol Chem (2018) 293:10895–910. doi: 10.1074/jbc.RA118.003650 PMC6052209 29880646

[191] Ong SC Belgi A Van Lierop B Delaine C Andrikopoulos S MacRaild CA. Probing the Correlation Between Insulin Activity and Structural Stability Through Introduction of the Rigid A6–A11 Bond. J Biol Chem (2018) 293:11928–43. doi: 10.1074/jbc.RA118.002486 PMC6066309 29899115

[192] Dubot P Boisseau N Cenedese P. Large Scale Full QM/MD Investigation of Small Peptides and Insulin Adsorption on Ideal and Defective TiO_2_ (1 0 0) Surfaces. Influence of Peptide Size on Interfacial Bonds. Appl Surface Sci (2018) 440:614–26. doi: 10.1016/j.apsusc.2018.01.190

[193] Macháčková K Mlčochová K Potalitsyn P Hanková K Socha O Buděšínskỳ M. Mutations at Hypothetical Binding Site 2 in Insulin and Insulin-Like Growth Factors 1 and 2 Result in Receptor-and Hormone-Specific Responses. J Biol Chem (2019) 294:17371–82. doi: 10.1074/jbc.RA119.010072 PMC6873181 31558604

[194] Ryberg LA Sønderby P Bukrinski JT Harris P Peters GH. Investigations of Albumin–Insulin Detemir Complexes Using Molecular Dynamics Simulations and Free Energy Calculations. Mol Pharmaceut (2019) 17:132–44. doi: 10.1021/acs.molpharmaceut.9b00839 31790268

[195] Todorova N Yarovsky I. The Enigma of Amyloid Forming Proteins: Insights From Molecular Simulations. Aust J Chem (2019) 72:574–84. doi: 10.1071/CH19059

[196] Salar S Jafari M Kaboli SF Mehrnejad F. The Role of Intermolecular Interactions on the Encapsulation of Human Insulin Into the Chitosan and Cholesterol-Grafted Chitosan Polymers. Carbohydr Polymers (2019) 208:345–55. doi: 10.1016/j.carbpol.2018.12.083 30658810

[197] Shin E Joo SH Yeom MS Kwak SK. Theoretical Study on the Stability of Insulin Within Poly-Isobutyl Cyanoacrylate (PIBCA) Nanocapsule. Mol Simulat (2019) 45:896–903. doi: 10.1080/08927022.2019.1609671

[198] Salehi SM Koner D Meuwly M. Dynamics and Infrared Spectrocopy of Monomeric and Dimeric Wild Type and Mutant Insulin. J Phys Chem B (2020) 124:11882–94. doi: 10.1021/acs.jpcb.0c08048 33245663

[199] Palanisamy K Prakash M. The Molecular Mechanism Behind the Stabilization of Insulin by Choline and Geranate (CAGE) Ionic Liquids–Computational Insights Into Oral Insulin Drug Formulation. Phys Chem Chem Phys (2021) 23:25298–307. doi: 10.1039/D1CP03349B 34746944

[200] Koliński M Dec R Dzwolak W. Multiscale Modeling of Amyloid Fibrils Formed by Aggregating Peptides Derived From the Amyloidogenic Fragment of the A-Chain of Insulin. Int J Mol Sci (2021) 22:12325. doi: 10.3390/ijms222212325 34830214 PMC8621111

[201] Li C Ma Y Liu X Huang R Su R Qi W. Synergistic Effect of Polystyrene Nanoplastics and Contaminants on the Promotion of Insulin Fibrillation. Ecotoxicol Environ Saf (2021) 214:112115. doi: 10.1016/j.ecoenv.2021.112115 33691242

[202] Feng YH Zhang XP Li WX Guo XD. Stability and Diffusion Properties of Insulin in Dissolvable Microneedles: A Multiscale Simulation Study. Langmuir (2021) 37:9244–52. doi: 10.1021/acs.langmuir.1c01434 34301147

[203] Zwier MC Chong LT. Reaching Biological Timescales With All-Atom Molecular Dynamics Simulations. Curr Opin Pharmacol (2010) 10:745–52. doi: 10.1016/j.coph.2010.09.008 20934381

[204] Lindahl E. Molecular Dynamics Simulations. Mol Model Proteins (Springer) (2015) 1215:3–26. doi: 10.1007/978-1-4939-1465-4_1 25330956

[205] Ayton GS Noid WG Voth GA. Multiscale Modeling of Biomolecular Systems: In Serial and in Parallel. Curr Opin Struct Biol (2007) 17:192–8. doi: 10.1016/j.sbi.2007.03.004 17383173

[206] Amaro RE Mulholland AJ. Multiscale Methods in Drug Design Bridge Chemical and Biological Complexity in the Search for Cures. Nat Rev Chem (2018) 2:1–12. doi: 10.1038/s41570-018-0148 PMC6445369 30949587

[207] Mieres-Perez J Sanchez-Garcia E. Quantum Mechanics/Molecular Mechanics Multiscale Modeling of Biomolecules. Adv Phys Organic Chem (Elsevier) Vol. (2020) 54:143–83. doi: 10.1016/bs.apoc.2020.08.002

[208] Visscher KM Geerke DP. Deriving a Polarizable Force Field for Biomolecular Building Blocks With Minimal Empirical Calibration. J Phys Chem B (2020) 124:1628–36. doi: 10.1021/acs.jpcb.9b10903 PMC7061328 32073849

[209] Huang J Lopes PE Roux B MacKerell AD Jr. Recent Advances in Polarizable Force Fields for Macromolecules: Microsecond Simulations of Proteins Using the Classical Drude Oscillator Model. J Phys Chem Lett (2014) 5:3144–50. doi: 10.1021/jz501315h PMC4167036 25247054

[210] Ren P Ponder JW. Polarizable Atomic Multipole Water Model for Molecular Mechanics Simulation. J Phys Chem B (2003) 107:5933–47. doi: 10.1021/jp027815+

[211] Jumper J Evans R Pritzel A Green T Figurnov M Ronneberger O. Highly Accurate Protein Structure Prediction With AlphaFold. Nature (2021) 596:583–9. doi: 10.1038/s41586-021-03819-2 PMC8371605 34265844

[212] Baek M DiMaio F Anishchenko I Dauparas J Ovchinnikov S Lee GR. Accurate Prediction of Protein Structures and Interactions Using a Three-Track Neural Network. Science (2021) 373:871–6. doi: 10.1126/science.abj8754 PMC7612213 34282049

[213] Vashisth H Abrams CF. Docking of Insulin to a Structurally Equilibrated Insulin Receptor Ectodomain. Proteins (2010) 78:1531–43. doi: 10.1002/prot.22670 20112420

[214] Vashisth H Maragliano L Abrams CF. “DFG-flip” in the Insulin Receptor Kinase is Facilitated by a Helical Intermediate State of the Activation Loop. Biophys J (2012) 102:1979–87. doi: 10.1016/j.bpj.2012.03.031 PMC3328698 22768955

[215] Vashisth H Abrams CF. All-Atom Structural Models of Insulin Binding to the Insulin Receptor in the Presence of a Tandem Hormone-Binding Element. Proteins (2013) 81:1017–30. doi: 10.1002/prot.24255 23348915

[216] Vashisth H. Flexibility in the Insulin Receptor Ectodomain Enables Docking of Insulin in Crystallographic Conformation Observed in a Hormone-Bound Microreceptor. Membranes (2014) 4:730–46. doi: 10.3390/membranes4040730 PMC4289863 25309993

[217] Mohammadiarani H Vashisth H. Insulin Mimetic Peptide S371 Folds Into a Helical Structure. J Comput Chem (2017) 38:1158–66. doi: 10.1002/jcc.24746 28190265

[218] Gorai B Vashisth H. Structures and Interactions of Insulin-Like Peptides From Cone Snail Venom. Proteins (2022) 90:680–90. doi: 10.1002/prot.26265 PMC8816879 34661928

